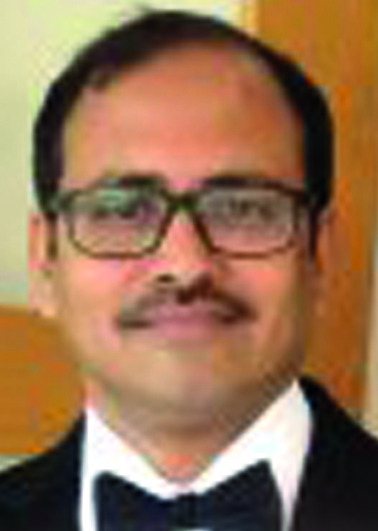# Nanocellulose Extraction from Biomass Waste: Unlocking Sustainable Pathways for Biomedical Applications

**DOI:** 10.1002/tcr.202400249

**Published:** 2025-03-04

**Authors:** Mehrdad Ghamari, Suvish, Chan Hwang See, Hongnian Yu, Thiyagarajan Anitha, V. T. Balamurugan, Sasireka Velusamy, David Hughes, Senthilarasu Sundaram

**Affiliations:** ^1^ Cybersecurity and Systems Engineering School of Computing, Engineering and the Built Environment Edinburgh Napier University Merchiston Campus Edinburgh EH10 5DT United Kingdom; ^2^ School of Computing, Engineering and Digital Technologies Teesside University Tees Valley Middlesbrough TS1 3BX United Kingdom; ^3^ Department of Postharvest Technology Horticultural College and Research Institute Periyakulam, Theni, Tamil Nadu 625 604 India; ^4^ Department of Biomedical Engineering Bannari Amman Institute of Technology Sathya Mangalam, Theni, Tamil Nadu 638 402 India

**Keywords:** Nanocellulose production, Waste biomass sources, Biomedical applications, Pretreatment methods, Electrochemical biosensors

## Abstract

The escalating global waste crisis necessitates innovative solutions. This study investigates the sustainable production of nanocellulose from biomass waste and its biomedical applications. Cellulose‐rich materials–including wood, textiles, agricultural residues, and food by‐products–were systematically processed using alkaline, acid, and oxidative pretreatments to enhance fiber accessibility. Mechanical techniques, such as grinding and homogenization, combined with chemical methods like acid hydrolysis and 2,2,6,6‐Tetramethylpiperidin‐1‐yl‐oxyl (TEMPO) oxidation, were employed to successfully isolate nanocellulose. Post‐treatment modifications, including surface coating and cross‐linking, further tailored its properties for specific applications. The results demonstrated nanocellulose's biocompatibility, biodegradability, and functional versatility. In wound healing, it enhanced moisture management and exhibited antimicrobial properties. Its high surface area facilitated efficient drug loading and controlled release in drug delivery applications. Nanocellulose bioinks supported cell proliferation in 3D bioprinting for tissue engineering. Additional applications in biosensors and personal care products were also identified. This study advances sustainable materials science, aligning resource conservation with circular economy principles to address biomedical sector needs.

## Graphical abstract

Sustainable extraction methods for converting diverse biomass waste into nanocellulose with remarkable biomedical properties were explored. Optimized pretreatment, mechanical processing, and surface modification techniques that enhanced the functionality of nanocellulose were detailed. The extracted nanomaterial demonstrated exceptional performance in wound healing, drug delivery, tissue engineering, and biosensor applications, representing a promising circular economy solution for healthcare innovation′′

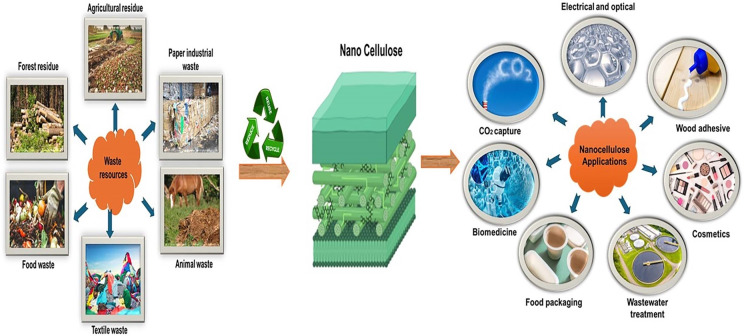

## 1 Introduction

The escalating issue of waste generation has become a paramount global concern due to its profound environmental implications. Improper waste disposal practices contribute to pollution, resource depletion, and climate change necessitating a comprehensive waste management approach that includes recycling and embraces circular economy principles.^[1]^ This research delves into the material characteristics and production process of nanocellulose from waste materials, exploring how this innovative application aligns with sustainable practices and circular economy principles.

Waste generation poses significant environmental challenges worldwide. The indiscriminate disposal of waste not only results in the depletion of natural resources but also contributes to environmental pollution and climate change. The United Nations Environment Programme highlights the severity of the issue, emphasizing the need for effective waste management strategies to mitigate these environmental effects. Addressing this challenge requires a holistic approach that incorporates recycling and embraces the principles of a circular economy, focusing on minimizing waste through continuous resource reuse.^[2]^

In the United Kingdom, the government actively prioritizes waste recycling and management as part of its commitment to environmental sustainability.^[3]^ The financial commitment to waste management initiatives underscores the importance placed on addressing this critical issue. Public expenditure on waste management in the United Kingdom reached 10.2 billion British pounds in 2022/23.^[4]^ An overall target to eliminate avoidable waste and double resource productivity by 2050 underpins the detailed plan.

The process of recycling, a key component of sustainable waste management, involves a series of systematic steps. These include collection, sorting, processing and the transformation of waste materials into new products.^[5]^ Efficient recycling begins with separate collection systems for different types of waste, such as plastics and paper. Advanced sorting technologies, incorporating both automated and manual techniques, facilitate effective separation at materials recovery facilities. The sorted waste materials undergo processing, which may include shredding, grinding, melting or chemical treatment, depending on the material type. The resulting secondary raw materials are then remanufactured and distributed as recycled goods, completing the recycling loop.^[6]^ The circular economy concept emerges as a powerful strategy to significantly reduce waste by promoting a “closed‐loop” approach to resource use.^[7]^ This approach emphasizes the continuous reuse of products and the recovery of materials at the end of their life cycle. Strategies such as designing products for durability, repairability and recyclability contribute to extending the lifespan of materials.^[8]^ Extended producer responsibility policies further incentivize waste reduction by holding manufacturers accountable for sustainable product lifecycles and recyclability.^[9]^ By minimizing material leakage from the system, circular economy principles achieve higher resource productivity.^[10]^ Transitioning to a circular economy offers transformative solutions beyond traditional recycling methods. Circular systems prioritize the regeneration of materials, keeping them in use for longer periods.^[11]^ This approach not only reduces the environmental impact of waste but also creates economic opportunities by turning waste into valuable resources.^[12]^

**Figure 1:**
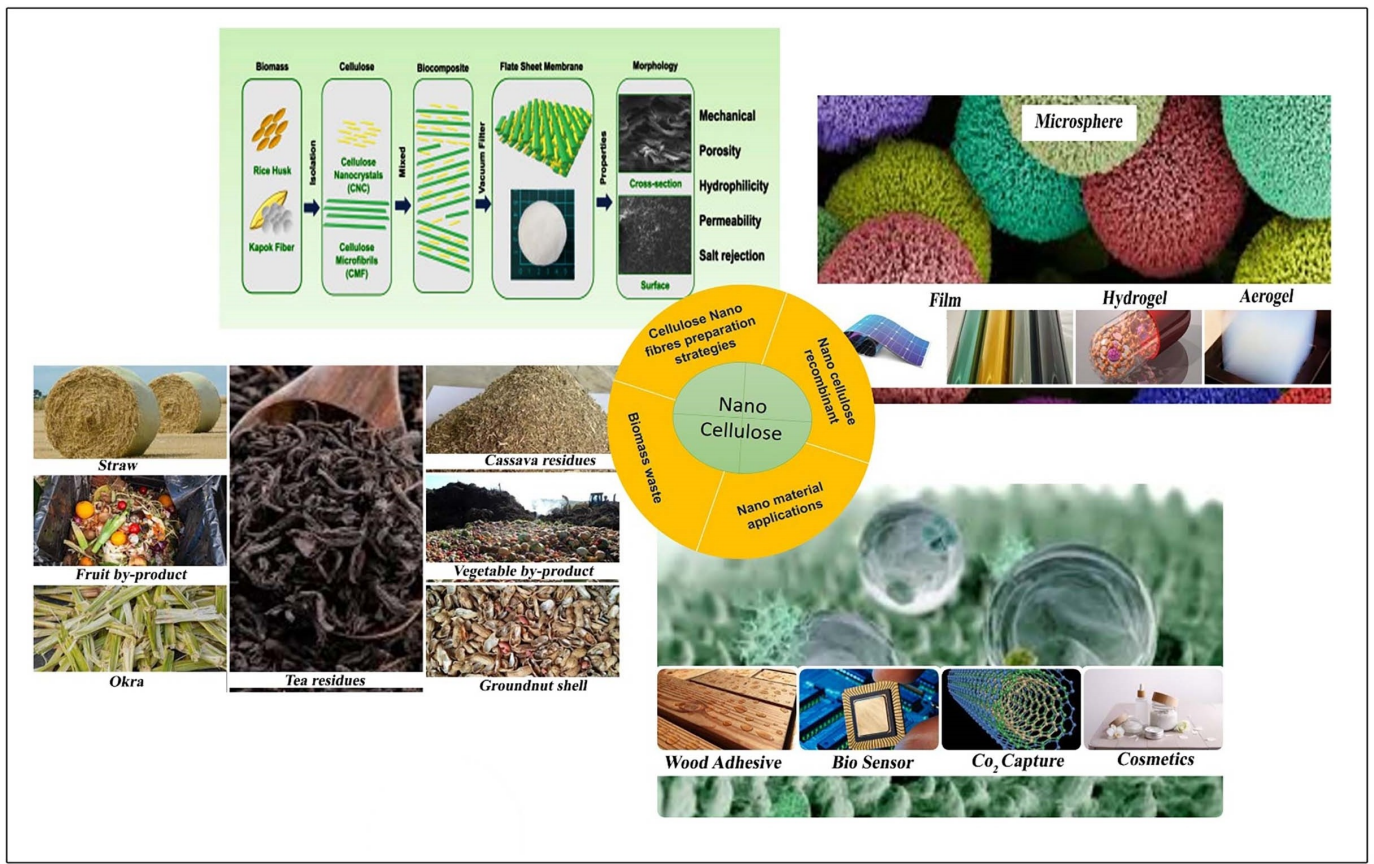
Schematic Representation of Nanocellulose Extraction from Biomass Waste for Sustainable Biomedical Applications.

The transformation of waste resources into nanocellulose stands as a remarkable illustration of the application of circular economy principles, marking a significant stride toward more sustainable and circular futures. Nanocellulose, a nanomaterial derived from cellulose, the main component of plant cell walls, boasts distinctive properties that render it highly versatile across various industries, including packaging, construction, healthcare and electronics. Abundant sources of cellulose fibers, present in waste wood,^[13]^ textile,^[14]^ paper,^[15]^ agricultural residues,^[16]^ animal,^[17]^ and food byproducts,^[18]^ provide an opportunity to harness these underutilized waste streams for nanocellulose production. The process involves a series of steps, starting with fiber extraction and subsequent processing, ultimately transforming these waste materials into a valuable nanomaterial. Figure 1 presents a schematic overview of the nanocellulose extraction process from biomass waste, detailing key stages from raw material selection to final production for sustainable biomedical applications.

**Figure 2:**
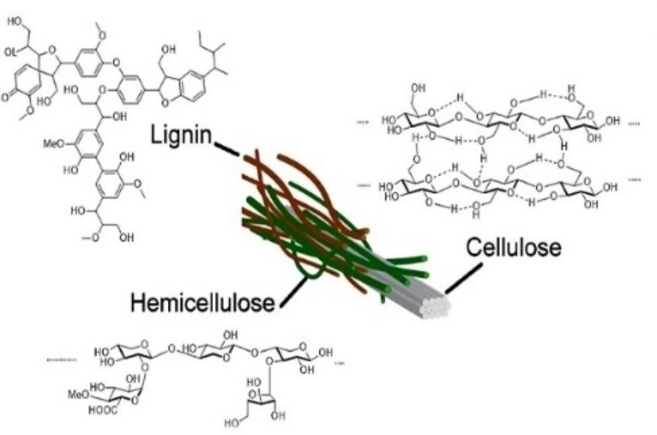
Molecular Structures of Cellulose, Hemicellulose, and Lignin.[22] Copyright 2024, Elsevier Ltd.

The structure of biomass waste is characterized by a complex interplay of three primary components: cellulose, hemicellulose, and lignin, which together form the sophisticated architecture of plant cell walls. The interaction of these components, which are integral to the architecture of plant cell walls, is highlighted as an essential element in comprehending and refining the production of nanocellulose from sustainable origins. Cellulose delivers structural integrity by forming microfibrils,^[19]^ hemicellulose improves flexibility,^[20]^ and lignin serves as a binding matrix, ensuring structural rigidity and providing protection against microbial degradation.^[21]^ This biomass complexity makes it valuable for sustainable nanocellulose production. Figure 2 shows the molecular structures of cellulose, hemicellulose, and lignin, highlighting their roles as key components of plant cell walls.

Nanocellulose's versatility extends its application across various industries, particularly benefiting the healthcare sector through biodegradable materials for diverse biomedical applications and personal care products. In wound healing, nanocellulose‐based dressings excel in moisture management, oxygen permeability, and mechanical strength, facilitating effective wound healing.^[23]^ Functionalization with antimicrobial agents or growth factors enhances nanocellulose's potential to prevent infections and expedite the healing process.^[24]^

In the context of pharmacological administration, the substantial surface area and tunable surface characteristics of nanocellulose facilitate efficient drug encapsulation and enable meticulous regulation of release kinetics, thereby enhancing therapeutic efficacy while mitigating adverse effects.^[25]^ Functionalization with targeting moieties further improves specificity and reduces off‐target effects.^[26]^

Waste‐derived nanocellulose, with its unique properties, has emerged as an attractive material for 3D‐bioprinting, biosensing and cosmetic applications. In tissue engineering, nanocellulose‐based bioinks offer structural support, mimicking the extracellular matrix and promoting cell adhesion, proliferation, and differentiation. These bioinks, combined with growth factors, cells, or other biomolecules, create intricate 3D structures resembling native tissues or organs.^[27]^ For biosensing applications, nanocellulose‐based electrodes or optical sensors, when functionalized with specific biomolecules, enable the detection and quantification of various analytes, thereby enhancing diagnostic capabilities.^[28]^ Additionally, nanocellulose's outstanding water‐holding capacity, biocompatibility and film‐forming properties make it well‐suited for cosmetic formulations like moisturizers, anti‐aging creams, and sunscreens.^[29]^

The shift towards a more circular and sustainable future requires creative solutions that integrate waste management with materials development. Converting waste resources into nanocellulose stands as a testament to the possibilities within the circular economy framework. As industries increasingly adopt such innovative practices, the potential for reducing waste, conserving resources, and minimizing environmental impact becomes more tangible. The journey towards sustainable and circular futures is propelled by initiatives that turn waste into valuable resources and nanocellulose production represents a promising and transformative step in this direction.

**Figure 3:**
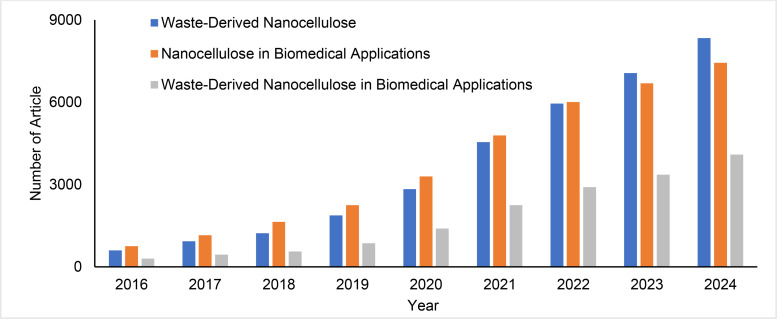
Annual Publication Trends of Nanocelluloses and Waste‐Derived Nanocelluloses in Biomedical Applications (2015 to March 12, 2024).

The analysis of annual publication trends is essential for comprehending the dynamic landscape of scientific research. Figure 3 in this study presents a thorough overview of yearly publications concerning waste‐derived nanocelluloses and the utilization of nanocellulose in biomedical applications from 2015 to March 12, 2024. The data for this examination were meticulously gathered from reliable sources, specifically the Web of Science Core Collection and Scopus, ensuring a robust and comprehensive representation of research output. In 2015, there were 416 publications on waste‐derived nanocelluloses, 535 on nanocelluloses, and 203 on waste‐derived nanocelluloses in biomedical applications. Subsequent years exhibited a consistent and gradual increase, reaching significant figures in 2023, with 6709, 6402 and 3181 publications on waste‐derived nanocelluloses, nanocelluloses, and waste‐derived nanocelluloses in biomedical applications, respectively. These statistics highlight a heightened level of interest and emphasis within the research community on these innovative technologies, underscoring their increasing significance in the respective scientific field.

## 2 Utilizing Waste Materials for Nanocellulose Production

**Figure 4:**
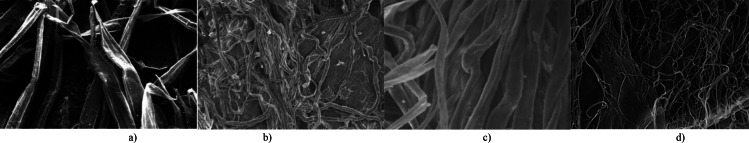
SEM image of different types of nanocellulose from Various Biomass Sources. a) CMF from hemp stalks (Scale:100 um), b) CNF from pineapple leaves (Scale:100 um), c) CNC from Waste cotton cloth (Scale:100 um), d) BNC from Acetobacter (Scale:5 μm).[62] Copyright 2024, Elsevier Ltd.

Nanocellulose refers to cellulose‐based materials with at least one dimension in the nanoscale.^[30]^ It can be derived from various cellulose‐rich waste materials, offering an innovative solution for sustainable resource use and waste management. Nanocellulose boasts unique strengths like high strength, biodegradability, and versatility, lending itself to diverse applications. The production process comprises pretreatment, cellulose extraction, nanocellulose synthesis via mechanical or chemical routes.^[31]^ Figure 4 shows various types of nanocellulose from different biomass sources, as imaged by scanning electron microscopy (SEM). Nanocellulose is categorized according to its dimensional structure, formation process, and key properties, as outlined in Table 1.

**Table 1:** Nanocellulose Types: Characteristics, Formation, and Applications.

Nano‐Cellulose	Diameter	Length	Shape	Surface area [m^2^ G−1]	Crystallinity	Young's Modulus (GPa)	Density (g cm^−3^)	Main Characteristic	Applications
Cellulose microfibers (CMF)	10 mm^[32]^	>10 mm.^[32]^	Long, slender fibers^[32]^	<1 e^6[33]^	51–69 %^[34]^	20^[33]^	0.6–0.7^[35]^	High aspect ratio.^[32]^	Reinforcement,^[36]^ composites,^[37]^ textiles,^[38]^ biomedical.^[39]^
Cellulose Nanofibrils (CNF)	5–60 nm^[40]^	Several micrometers.^[40]^	Fiber shape^[41]^	~100^[33]^	45–80 %^[42]^	50–160^[33]^	1.5^[40]^	High aspect ratio, Biodegradable, High strength.^[43]^	Paper products,^[44]^ Food packaging,^[45]^ Rheological modifiers,^[46]^ Biocomposites.^[47]^
Cellulose Nanocrystalline (CNC)	5–70 nm^[40]^	100–250 nm.^[40]^	Rodlike shape^[41]^	~200^[33]^	54–88 %^[42]^	50–140^[33]^	1.6^[40]^	High crystallinity, Reinforcing agent, Biocompatible.^[48]^	Reinforcement in composites,^[49]^ Drug delivery,^[50]^ Biomedical applications,^[51]^ Coatings.^[52]^
Bacterial Nano‐Cellulose (BNC)	20–100 nm^[40]^	several micrometers.^[53]^	Fibrous^[54]^	~1500^[33]^	50–60 %^[55]^	16.4^[56]^	14.7^[57]^	Pure and ultrafine fibers, Biocompatible, water holding capacity.^[58]^	Wound dressings,^[59]^ Tissue engineering,^[60]^ Food additives.^[61]^

## 3 Manufacture Techniques

**Figure 5:**
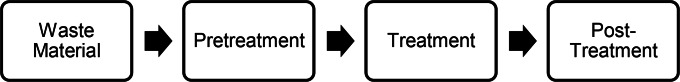
Nanocellulose Production Process.

The process of making nanocellulose encompasses several crucial steps: Raw Material Selection, Pre‐treatment, Treatment and Post‐Treatment Process, as illustrated in Figure 5. In this structured sequence, raw materials are meticulously chosen based on specific attributes. The pre‐treatment phase focuses on removing impurities and enhancing accessibility in cellulose fibers. Subsequently, the treatment step employs various methods such as bleaching and acid hydrolysis to modify the cellulose. The final stage involves post‐treatment processes to refine the nanocellulose, ensuring it meets the desired specifications for intended applications.

The ensuing discourse will delve into a comprehensive and detailed review of the various stages involved in the production of diverse types of nanocellulose sourced from additional materials. Each production stage, from raw material selection to quality control, will be meticulously scrutinized in the context of nanocellulose derived from materials beyond those initially outlined. This examination aims to provide a nuanced understanding of the intricacies and optimizations inherent in the manufacturing processes, elucidating the unique attributes originating from a broader spectrum of source materials.

### 3.1 Select Raw Materials

Table 2 in the academic publication underscores the sourcing of nanocellulose, accentuating the pivotal function of waste materials in facilitating sustainable nanomaterial production. It analyzes significant factors such as cellulose, hemicellulose, and lignin content, which are critical for ascertaining the characteristics of nanocellulose obtained from diverse waste streams, encompassing wood, textiles, paper, agriculture, and food waste.

**Table 2:** Comparative Analysis of Nanocellulose Properties from Waste Sources.

Waste Material		Cellulose	Hemicellulose	Lignin
Textile^[63]^	Industrial cotton waste, Bombyx mori silk.	60–90 %	3–6 %	1–15 %
Paper^[64]^	Wood flour, Secondary Paper Waste, Waste Newspaper, Waste Board and Milk Container Board, Mixture of Cardboard and Newspaper Wastes, Flax Fibers.	60–70 %	10–20 %	5–10 %
Agricultural[^65^, ^66^]	Sugarcane Bagasse, Pineapple Leaf, Wheat Straw, Corn Stover, Rice Straw, Coconut Husk fiber, Kenaf Hemp.	60–80 %	5–15 %	5–25 %
Wood^[67]^	Sawdust, Bamboo, Jute, Eucalyptus.	40–45 %	25–30 %	15–30 %
Animal^[68]^	Cow Dung, Horse Dung, Elephant Dung.	1–25 %	1–15 %	2–15 %
Food^[69]^	Tomato Peel, Banana, Soybean Pods, Orange Waste, Oil Palm Biomass Residue, Carrot Residue.	10–65 %	5–35 %	1–40 %

### 3.2 Pretreatment

**Figure 6:**
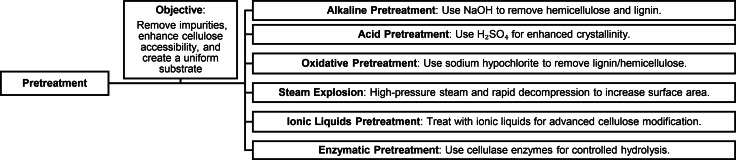
Pretreatment Strategies for Nanocellulose Production.

The pretreatment phase is a critical and essential stage in the complex process of producing nanocellulose from cellulose fibers. Its primary objectives include the precise removal of impurities, the improvement of cellulose fiber accessibility, and the establishment of a uniform substrate conducive to subsequent hydrolysis.^[70]^ During the pretreatment of plant materials, a focused elimination of non‐cellulose components, particularly hemicellulose and lignin, is meticulously carried out, resulting in the isolation of individual fibers.^[71]^ In the realm of BNC, the pretreatment process demonstrates notable efficacy in eliminating bacteria and impurities from the slurry, contributing to the production of high‐quality nanocellulose.^[72]^ Figure 6 outlines the steps for pretreating waste‐derived nanocellulose. It includes various methods alkaline, acid, oxidative, steam explosion, ionic liquids, and enzymatic—along with criteria for evaluating effectiveness.

There are several pre‐treatment methods that can be used to prepare cellulose fibers for nanocellulose production, including:

- The alkaline pretreatment is a pivotal step wherein cellulose fibers undergo treatment with a potent alkali solution, such as sodium hydroxide, aiming to eliminate hemicellulose and lignin.^[73]^ This process holds the promise of augmenting the surface area of cellulose fibers, thereby improving their accessibility to hydrolysis agents. However, caution is warranted as excessive alkali treatment may precipitate cellulose degradation, potentially compromising the quality of nanocellulose. Notably, alkaline treatment has demonstrated a pronounced capacity to enhance the purity and crystallinity of cellulose, leading to an elevated yield of nanocellulose.^[74]^
- Acid Pre‐treatment: In the acid pre‐treatment method, cellulose fibers undergo treatment with a potent acid, such as sulfuric acid, aiming to remove hemicellulose and lignin. This process has the potential to enhance the crystallinity of cellulose fibers, thereby improving the overall quality of nanocellulose. However, it is crucial to note that acid pre‐treatment comes with potential drawbacks, including the risk of cellulose degradation and a subsequent decrease in the yield of nanocellulose. One specific application of acid pre‐treatment is in the production of CNC from cellulose fibers. Through acid hydrolysis, typically using strong acids like sulfuric acid, this process results in the formation of highly crystalline and rod‐shaped CNC. The breakdown of cellulose fibers into smaller particles during acid hydrolysis contributes to an increased surface area and improved accessibility of the cellulose, which are essential factors in the nanocellulose production process.^[75]^
- Oxidative pretreatment: is a method that employs an oxidizing agent, such as sodium hypochlorite, to selectively remove lignin and hemicellulose from cellulose fibers. This treatment results in a notable increase in the surface area, accessibility, and reactivity of cellulose fibers. By utilizing oxidative agents, the process modifies the chemical composition of the fibers, effectively breaking down lignocellulosic bonds. This, in turn, enhances the overall properties of cellulose, making it more amenable to subsequent processing steps. The oxidative pre‐treatment is particularly valuable in nanocellulose production, where improved surface characteristics and reactivity contribute to the efficiency and quality of the final nano cellulosic material.[^76^, ^77^]
- Steam explosion, a significant pretreatment method for nanocellulose production, involves subjecting cellulose fibers to high‐pressure steam followed by rapid decompression. This process increases the accessibility and surface area of cellulose fibers, resulting in a notable upsurge in the yield of nanocellulose. Notably, steam explosion pretreatment is found to reduce the overall energy demand of the production process, highlighting its dual advantages of enhanced nanocellulose yield and improved energy efficiency. This positions steam explosion as a valuable pretreatment method, especially when dealing with specific cellulose sources such as rice straw.[^78^, ^79^]
- Ionic Liquids Pretreatment: characterized as organic salts with a liquid state at room temperature, are employed in this process to treat cellulose fibers. Despite the effectiveness of ionic liquids, their application presents challenges, mainly due to their high cost and the difficulty in completely removing them from nanocellulose, which can result in contamination concerns. As such, while offering advantages in terms of product quality, the use of ionic liquids requires careful consideration of the associated economic and purification challenges.[^80^, ^81^]
- Enzymatic Pretreatment: is a specialized pre‐treatment method in nanocellulose production that utilizes cellulase enzymes to catalyze the hydrolysis of cellulose fibers. This process involves breaking down the long cellulose chains into smaller particles, thereby increasing the surface area and accessibility of the cellulose. The enzymatic treatment is particularly effective in selectively degrading cellulose without causing extensive damage to the fibers, resulting in well‐dispersed and highly reactive nanocellulose. The use of cellulase enzymes ensures a controlled and environmentally friendly approach to fiber modification. While specific details regarding the enzymatic treatment process may vary based on the cellulase source and experimental conditions, the overarching goal is to optimize the enzymatic action for improved nanocellulose characteristics.[^82^, ^83^]

The choice of an appropriate pretreatment method is crucial and depends on the specific characteristics of the starting material and the desired properties of the final nanocellulose product. Effective pretreatment methods play a vital role in significantly reducing energy consumption during nanocellulose production. This reduction in energy usage can be substantial, with reported decreases of 20–30 times through efficient pretreatment.^[84]^ The integration of mechanical, chemical, or enzymatic methods, significantly reduces overall energy consumption in nanocellulose production.^[85]^ The positive effects of proper pretreatment on cellulose fiber properties, including enhanced accessibility, increased inner surface area, altered crystallinity, hydrogen bond breakage, and boosted reactivity, collectively contribute to a noteworthy decrease in the overall energy demand throughout the nanocellulose production process.[^86^, ^87^]

### 3.3 Treatment

**Figure 7:**
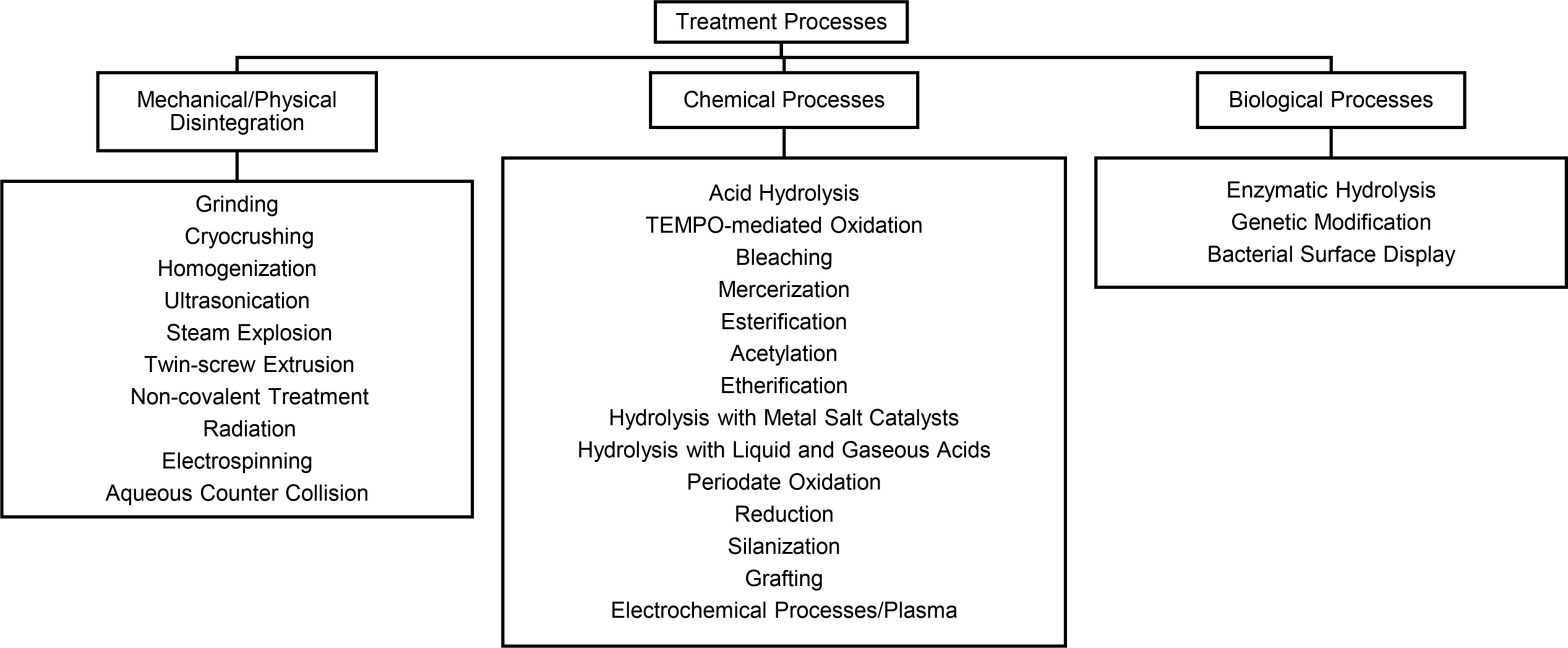
Classification of Treatment for Nanocellulose Production

The treatment process in nanocellulose production is a meticulously designed sequence of steps aimed at refining cellulose fibers into nanoscale dimensions and tailoring their properties for diverse applications. Figure 7 presents a hierarchical overview of nanocellulose production methods, categorizing them into three main types: mechanical/physical disintegration, chemical processes, and biological processes. Each category includes various specific techniques.

#### 3.3.1 Mechanical/Physical Disintegration

Mechanical disintegration of cellulose fibers utilizes high‐shear forces such as grinding, cryocrushing, homogenization, ultrasonication, steam explosion, and twin‐screw extrusion (TSE) to break down cellulose structures and release nanocellulose. This process achieves nanoscale dimensions, enhancing material properties for diverse applications. As a crucial step in nanocellulose production, mechanical disintegration lays the groundwork for further refinement and customization, ensuring suitability for specific industrial uses, including biomedical, packaging, and composite material applications.

##### 3.3.1.1 Grinding

Grinding is an effective mechanical process to disintegrate cellulose fibers into nanoscale structures. Studies on maize stalk‐derived nanocellulose showed grinding produced CNF of 4–10 nm diameter and micrometer lengths. CNC of 3–7 nm diameter, 150–450 nm length, and high 50–64 aspect ratios were also obtained.^[88]^ Grinding decreased CNF crystallinity from 70.5 % to 66.4 % by disrupting crystalline regions. Lower crystallinity improved nanopaper transparency compared to CNC but reduced thermal stability versus conventional cellulose. CNF web shapes enabled stress transfer between fibrils, enhancing mechanical properties. Further works studied grinding's impacts on various cellulosic sources. Acid hydrolysis with grinding generated 40–80 nm diameter CNFs from wheat straw.^[89]^ Bleached eucalyptus pulp produced 5–30 nm, or 5–20 nm diameter CNFs without and with enzymatic pretreatment respectively.^[90]^ However, uneven grinding caused partial fibril separation and twisting. Optimizing treatment extent, fibrillation level, and preserving aspect ratio are key for stable rheology and performance.^[91]^

##### 3.3.1.2 Cryocrushing

Cryocrushing represents a prevalent technique for the synthesis of nanofibers, wherein cellulose fibers are subjected to freezing via liquid nitrogen followed by mechanical crushing under elevated shear forces. The freezing mechanism induces the formation of ice crystals that apply pressure, thereby disrupting cell walls and yielding microfibrils. When coupled with high‐pressure fibrillation, this methodology proficiently segregates nanofibers from diverse raw materials.^[92]^ Cryocrushing not only embrittles cellulose, reducing the mechanical force needed for size reduction, but also sets the stage for subsequent defibrillation, where microfibrils are detached into nanofibers. This method enhances nanocellulose properties and facilitates scalable production by providing an efficient preparatory step before disintegration. For instance, soybean fibers treated with cryocrushing, followed by 20 passes of 500–1000 bar defibrillation, yielded nanofibers measuring 50–100 nm in width and micrometers in length.^[93]^ Cryo milled wheat straw fibers, processed through a disintegrator, resulted in nanofibers with diameters of 30–40 nm.^[94]^ The subsequent defibrillation process fluidizes the ruptured cell wall fragments, aiding in the separation of nanofibers.^[95]^

##### 3.3.1.3 Homogenization

High‐pressure homogenization has emerged as an effective method for the production of cellulose nanoparticles (CNP), including CNFs and CNC, from various cellulose sources.[^96^, ^97^] Acid hydrolysis pretreatment, followed by mechanical defibrillation through high‐pressure homogenization, enables the nanoscale fibrillation of cellulose. Several factors, including the acid type, concentration, hydrolysis duration, and the number of homogenization cycles, play a crucial role in determining the morphology and properties of the resulting nanoparticles.^[98]^ Regarding concentration, the concentration of the acid used in the hydrolysis process directly impacts the efficiency of cellulose breakdown. A higher acid concentration typically accelerates the hydrolysis, leading to more effective fibrillation, but it can also influence the structural characteristics of the nanoparticles, such as their size, surface area, and stability. A balanced concentration is essential to achieve the desired nanoparticle properties while preventing excessive degradation of the cellulose.^[99]^ For example, the production of CNF from microcrystalline cellulose (MCC) using a homogenizer at 20,000 psi with 0–20 passes. Transmission electron microscopy confirmed complete fibrillation at the nanoscale, with 5–10 passes producing reinforcing nanofibrils for hydroxypropyl cellulose films.^[100]^ Furthermore, high‐pressure homogenized cellulose has shown potential in composites and material applications. The CNF produced using sulfuric acid hydrolysis and homogenization exhibited high mechanical strength, optical transparency and surface area.^[101]^ Thermogravimetric analysis revealed variations in thermal stability based on acid type, attributed to surface chemistry changes like sulfate group introduction.^[102]^ Despite reduced thermal stability with sulfuric acid, the highly charged, high aspect ratio nanofibrils were considered suitable for advanced nanocomposites. The isolation of cellulose with tailored needle‐shaped morphology and high surface area from tomato pomace using sequential hydrolysis and 80 MPa 10‐pass homogenization highlighted the biorefinery potential of agri‐food wastes for cellulose and value‐added coproducts.^[96]^

##### 3.3.1.4 Ultrasonication

Ultrasonication has emerged as a promising method for the production of nanocellulose, including CNC and CNFs, from cellulosic sources like wood pulp and MCC.^[103]^ High‐intensity ultrasonication leads to the nanoscale fragmentation of cellulose fibers, resulting in rod‐shaped CNC between 10–20 nm wide and 50–250 nm long or fibrillar CNFs.^[104]^ Scanning and transmission electron microscopy confirm the morphology. X‐ray diffraction reveals some decline in crystallinity with longer sonication but retention of the cellulose I structure. Thermogravimetric analysis indicates slightly reduced thermal stability of nanocellulose compared to MCC, along with higher char residue.^[105]^

Furthermore, ultrasonication shows potential as a pretreatment to enhance acid hydrolysis yields and kinetics in nanocellulose production.^[106]^ Here, short ultrasonication treatments induced surface erosion and cracks on cellulose fibers, increasing accessibility for acid attack during hydrolysis. Response surface modeling helped optimize temperatures, acid concentrations and times, achieving 56 % optimized yield of 360 nm nanocellulose.^[107]^ The effects depend on sonication duration, manifesting first as oval degraded regions on fibers before longitudinal cracks form. Prolonged exposure leads to particle agglomeration without nanometer sizes.^[103]^ The acid concentration plays a pivotal role in the process. Higher concentrations can expedite the hydrolysis reaction, but an optimal balance is necessary to avoid over‐etching or degradation of cellulose fibers. The acid concentration influences the degree of surface erosion and the formation of cracks, which, in turn, determine the efficiency of the overall hydrolysis process and the final size and morphology of the nanocellulose

Ultrasonication facilitates greener and more sustainable nanocellulose production methods, either as an extraction booster through acoustic effects or for dispersion and deagglomeration.^[108]^ It can reduce chemical usage, process times and energy input compared to conventional mechanical treatments. Beyond this, micro jetting provides sufficient energy for nano fibrillation, indicating the potential to completely replace mechanical processing. The resulting nanocellulose demonstrates enhanced properties like higher crystallinity and aspect ratio. To fully utilize ultrasonication, future efforts should focus on extending it towards primary extraction roles rather than just dispersion. Combining ultrasonication with emerging greener extraction approaches can lead to more robust and sustainable nanocellulose production.^[106]^

##### 3.3.1.5 The Steam Explosion

Steam explosion is a successful method for producing CNC from lignocellulosic residues and waste materials. When combined with acid hydrolysis or enzymatic treatments, it breaks down cellulose fibers into nanoscale dimensions, typically ranging from 5–100 nm in width and 600–2600 nm in length.^[109]^ Steam‐coupled acid treatment of pineapple leaf fibers generated nanofibrils of 5–60 nm width,^[110]^ while steam explosion with cellulase digestion of poplar wood produced 20–50 nm width nanocellulose.^[111]^ The high pressure and sudden decompression induce fragmentation, increasing accessibility for further chemical attack. This results in high crystalline cellulose content above 90 %^[112]^ along with the removal of non‐cellulosic components like lignin and hemicellulose.^[113]^

Furthermore, steam explosion facilitates more sustainable and energy‐efficient nanocellulose production. Combining with lignin sulfonation using 18 % sodium sulfite allows direct production of micro and CNF from *Posidonia oceanica* seaweed without extensive pretreatments.^[114]^ Nanopapers displayed a similar modulus between 4.6–6.1 GPa despite lower intensities of mechanical processing employed. Additionally, the high‐yield pulps generated enable reduced chemical consumption overall compared to conventional processes. Steam explosion of banana fibers resulted in 95 % cellulose content, increasing from 64 % in the raw material.^[113]^

The nanocellulose materials produced through the steam explosion method demonstrate favorable morphological and thermal properties, making them well‐suited for both composite and biomedical applications. The surface chemistry and crystallinity alterations induced by steam explosion contribute to enhanced thermal stability, leading to increased decomposition temperatures.^[113]^ Evaluation of chemical composition through X‐ray diffraction and Fourier‐transform infrared spectroscopy (FTIR) offers valuable insights into structural changes and the removal of non‐cellulosic components.^[115]^ The resulting web‐like nanofibrillar structure, coupled with a high aspect ratio, positions steam‐exploded cellulose as an ideal material for various biomedical applications, including tissue scaffolds, medical implants, and drug delivery systems. Moreover, these nanocellulose fibers exhibit properties conducive to biomedical engineering, presenting a versatile material platform for applications such as wound healing, drug delivery, and scaffold‐based tissue regeneration. The increased thermal stability and biocompatibility observed in steam‐exploded cellulose fibers open novel avenues for innovative solutions in the expanding fields of biomedical and bioengineering.

##### 3.3.1.6 Twin‐Screw Extrusion (TSE)

TSE enables the continuous production of nanocellulose by applying intense shear forces and high pressures simultaneously.^[116]^ This process facilitates the nanofibrillation of cellulose fibers suspended in water, even at concentrations of up to 50 %, by passing the slurry through specially designed screw elements.^[117]^ By optimizing parameters such as screw profile design, speed, temperature, and feed rate, it is possible to control the intensity of fibrillation in a single pass, leading to reduced energy consumption. Additionally, TSE induces significant physicochemical changes in cellulose; higher temperatures reduce crystallinity, while the shear forces and pressure modify the surface chemistry. This allows for precise control over the morphology and properties of the final nanocellulose product.^[118]^ he cellulose slurry's concentration plays an essential role in the fibrillation process. At higher concentrations, the fiber network becomes denser, which can require more intense shear forces and higher pressures to achieve effective nanofibrillation. Concentration affects the ease with which the fibers are separated, as well as the overall energy input needed for efficient processing. Optimal concentration is critical for balancing the yield, energy efficiency, and final properties of the nanocellulose.^[118]^

The properties achieved make TSE suitable for biomedical applications. For example, 25 wt % nanocellulose pastes were 3D printed with excellent accuracy into scaffolds with controlled architecture.^[119]^ Mechanical properties reached 13 GPa modulus, providing critical reinforcement for load‐bearing implants.^[117]^ Surface modification introduces carbonyl and carboxyl groups, enabling biomolecule conjugation for programmed drug delivery.^[120]^ Thermal processes reduce crystallinity as well, tuning degradation rates for temporary implants. Overall, TSE allows continuous nanocellulose production with properties tailored for reinforcement, bio integration, and biofunctionalization across diverse medical devices.

##### 3.3.1.7 Non‐Covalent Treatment

Non‐covalent modifications provide a flexible way to customize the surfaces of nanocellulose for biomedical applications. The physical adsorption method, which uses weak intermolecular forces such as van der Waals interactions and hydrogen bonding, allows molecules like surfactants and polymers to adhere to the nanocellulose surfaces. This technique effectively alters properties such as wettability and dispersibility while preserving the material's inherent characteristics.[^121^, ^122^] Electrostatic adsorption, a specific form of physical adsorption, harnesses opposite charges to attract molecules, leading to alterations in surface properties such as wettability and dispersibility.^[123]^ Significant enhancement in the dispersibility of CNC in water through surface modification with cetyltrimethylammonium bromide (CTAB). CTAB‐modified CNC achieved 50–100 % charge coupling, stable in ethanol, forming a chiral nematic liquid crystal at ~4 wt . %.^[124]^

Layer‐by‐layer assembly, involving the sequential deposition of oppositely charged molecules, offers a controlled approach to constructing multilayered coatings on nanocellulose surfaces. This technique allows precise adjustments to properties like wettability and dispersibility).[^125^, ^126^] Hydrophobic modification, introducing hydrophobic molecules like fatty acids, enhances surface compatibility with hydrophobic materials and improves mechanical properties.[^127^, ^128^] Polymer wrapping, achieved through physical or electrostatic interactions or covalent bonding, adds a thin polymer layer, altering properties like wettability and stability.^[129]^

##### 3.3.1.8 Radiation

Radiation treatment for cellulose involves the application of high‐energy ionizing radiation, such as gamma rays or electron beams. This process aims to break down cellulose fibers by generating reactive species like free radicals, facilitating subsequent processing. The environmental benefits of radiation pretreatment include its minimal generation of toxic waste and lower energy consumption compared to alternative methods. The breaking of glycosidic bonds, leading to the formation of reactive species and subsequent cleavage of cellulose chains, results in the reduction of crystallinity and molecular weight.^[130]^

The effectiveness of radiation on cellulose fibers depends on factors like radiation type and dose, moisture content, and the presence of chemicals. Higher radiation doses result in increased chain scission and significant molecular weight reduction. However, excessive radiation can lead to undesirable by‐products, such as carbonyl groups, impacting the quality of nanocellulose. Studies have demonstrated radiation's effectiveness in modifying cellulose properties, improving the mechanical and barrier properties of films, and enhancing compatibility with other materials.[^131^, ^132^]

The impact of gamma radiation on poly (caprolactone)/CNC composite films revealed reduced oxygen and carbon dioxide Opermeabilities, indicating improved barrier properties.^[133]^ Mechanical and permeability modifications in nanocellulose/methylcellulose composite films after gamma radiation treatment.^[134]^ These improvements were attributed to induced crosslinking and degradation of cellulose fibrils. Ultraviolet (UV) radiation was explored for creating supramolecular healable materials and functionalizing CNC.[^135^, ^136^, ^137^]

##### 3.3.1.9 Electrospinning

Electrospinning is a technique that uses a high‐voltage electric field to create fine fibers from a polymeric solution. For cellulose electrospinning, the cellulose is dissolved in solvents like NMMO/water, LiCl/DMAc, ionic liquids, or ethylene diamine.^[138]^ The solution is pumped through a syringe and subjected to an electric field, causing it to elongate into nanofibers that are collected on a grounded surface.^[139]^ Nanofibers with diameters ranging from 50–500 nm can be produced from cellulose derivatives like cellulose acetate, hydroxypropyl cellulose, HPMC, and CMC. Fiber properties depend on factors like molecular weight, degree of substitution, and solvent, with cellulose acetate being preferred for its ease of processing and ability to produce pure cellulose fibers after deacetylation.^[138]^

The resulting electrospun cellulose nanofiber matrices have shown promise for biomedical applications. They can match the morphological structure of the natural extracellular matrix more closely than other scaffold materials.^[140]^ Studies found electrospun cellulose scaffolds exhibit good biocompatibility and can support rapid cell proliferation.^[141]^ However, challenges remain in controlling fiber alignment and pore size.^[142]^

##### 3.3.1.10 Aqueous Counter Collision

The aqueous counter collision (ACC) method is a mechanical technique that uses high‐pressure water jets colliding at 200 MPa to break down cellulose fibers into nanofibers.[^143^, ^144^] It can be combined with a chemical treatment like TEMPO‐oxidation which partially oxidizes the cellulose, making it easier to disintegrate by ACC. Using a 2 hr TEMPO oxidation of hardwood pulp followed by 5 passes through the ACC nozzle produced CNF around 15–17 nm wide, with high transparency (~90 %) and birefringence. The properties depended on factors like pulp source (hardwood vs softwood) and oxidation time.^[145]^

Isolated CNF can be analyzed by atomic force microscopy (AFM) to determine mechanical properties such as Young's modulus. AFM 3‐point bending tests on nanocellulose from various sources have been conducted. The hardwood, softwood, bamboo, and cotton nanocellulose had comparable Young's moduli around 88–110 GPa. Accurate measurement requires careful determination of nanofiber geometry and deflection by AFM.^[146]^ The disintegration of amorphous calcium carbonate (ACC) occurs preferentially along certain crystalline planes whose interactions are weakest, producing nanoparticles with some hydrophobic character.[^147^, ^148^] The amphiphilic nature of ACC‐produced CNF, with distinct hydrophobic and hydrophilic faces, was investigated using carbohydrate‐binding module probes. Quantitative adsorption measurements revealed differences in hydrophobicity depending on the cellulose source.^[148]^

Table 3 summarizes the key details and findings for various mechanical and physical disintegration treatment methods used in nanocellulose production. It highlights each method's contributions, including their effects on nanocellulose properties and production efficiency.

**Table 3:** Summary of Mechanical and Physical Disintegration treatment Methods for Nanocellulose Production: Key Findings and Contributions.

Treatment Method	Key Findings
Grinding	– Produces CNF and CNC with varying diameters and lengths. – Reduces crystallinity, impacting properties like transparency and thermal stability.
Cryocrushing	– Uses liquid nitrogen to freeze and then crush cellulose fibers. – Yields nanofibers with diameters of 30–100 nm. – Enhances disintegration and defibrillation efficiency.
Homogenization	– High‐pressure homogenization produces CNF and CNC from various sources. – Acid pretreatment and homogenization affect morphology and properties. – Produces high‐strength, transparent nanofibers.
Ultrasonication	– Uses high‐intensity sound waves to fragment cellulose fibers. – Reduces crystallinity and can enhance acid hydrolysis efficiency.
Steam Explosion	– Applies steam and pressure to break down cellulose fibers. – Results in high‐crystallinity nanocellulose with widths of 5–100 nm. – More sustainable with reduced chemical use.
TSE	– Continuous production method using shearing and high pressure. – Allows control over fibrillation and morphology. – Suitable for biomedical applications with tailored properties.
Non‐Covalent Treatment	– Modifies nanocellulose surfaces through adsorption and layer‐by‐layer assembly. – Enhances properties like wettability and dispersibility
Radiation	– Uses high‐energy radiation to break down cellulose fibers. – Results in reduced crystallinity and molecular weight. – Effective in improving properties like barrier performance.
Electrospinning	– Produces fine cellulose fibers from dissolved cellulose solutions. – Results in nanofibers with diameters of 50–500 nm. – Promising for biomedical applications with good biocompatibility.
ACC	– Uses high‐pressure water jets to disintegrate cellulose fibers. – Can be combined with chemical treatments like TEMPO‐oxidation. – Produces CNF with high transparency and specific mechanical properties.

#### 3.3.2 Chemical Processes

Chemical processes play a pivotal role in producing nanocellulose, utilizing methods like acid hydrolysis, TEMPO‐mediated oxidation, and bleaching for primary isolation. These techniques provide effective pathways to derive nanocellulose directly from raw biomass. Additionally, secondary modifications such as mercerization, esterification, acetylation, etherification, hydrolysis with metal salt catalysts, hydrolysis with gaseous acids, periodate oxidation, reduction, silanization, and grafting allow targeted adjustments to nanocellulose properties post‐isolation. This versatility provides researchers with a powerful toolbox to customize nanocellulose surface chemistry and functionality for specific applications while preserving its bulk properties. The dynamic nature of these chemical processes contributes significantly to the adaptability and diverse applications of nanocellulose across industries.

##### 3.3.2.1 Acid Hydrolysis

The acid hydrolysis of cellulose, employing acids like H_2_SO_4_, HCl, oxalic acid, HBr, H_3_PO_4_, and HNO_3_, is a well‐established method for producing well‐defined CNC.[^149^, ^150^] During this process, amorphous portions and local inter‐filler contacts of cellulose undergo hydrolysis, leaving stable crystallites that can be isolated as rod‐like nanocrystalline particles. The resulting CNC, suspended in strong acid, is then diluted, washed, and subjected to mechanical methods like ultrasonication for disintegration. However, hydrolysis with H_2_SO_4_ introduces negatively charged sulfate ester groups on the CNC surface, affecting thermal stability. To mitigate this, neutralization with NaOH is proposed, impacting CNC dispersion and thermal stability.^[151]^

The sulfate groups introduced during H_2_SO_4_ hydrolysis accelerate cellulose degradation, limiting CNC’ thermal stability and usability in applications like nanocomposite reinforcement. Mineral acids other than H_2_SO_4_, such as H_3_PO_4_, have gained attention for cellulose hydrolysis. CNC obtained through H_3_PO_4_ hydrolysis exhibit flame resistance and higher thermal stability compared to those from H_2_SO_4_ hydrolysis.^[152]^ The effectiveness of enzymatic hydrolysis following H_3_PO_4_ in enhancing CNC yield and improving dispersion, crystallinity, and thermal stability, showcasing potential applications in biomedical fields. These studies emphasize the significance of acid hydrolysis conditions in tailoring CNC properties for diverse applications, including those in biomedical materials.[^153^, ^154^]

##### 3.3.2.2 TEMPO‐Mediated Oxidation

TEMPO‐mediated oxidation has become a prominent method for treatment of nanocellulose, owing to its high efficiency, selectivity, and mild reaction conditions.^[155]^ This technique involves the oxidation of nanocellulose using TEMPO and a co‐oxidant, such as NaClO or NaBr, resulting in the introduction of carboxylic acid groups. This modification significantly enhances the dispersibility and reactivity of nanocellulose in various matrices.^[156]^ TEMPO‐mediated oxidation to CNFs observing improved dispersion in water and enhanced mechanical properties in resulting composite films.^[157]^

In the realm of biomedical applications, TEMPO‐mediated oxidation demonstrates its effectiveness through various studies. This method can introduce carboxylate groups onto carboxylated CNC, augmenting their negative charge and stability in aqueous suspension.[^158^, ^159^] the rheological properties of cellulose nanofibril suspensions, revealing that surface modification with carboxylate groups significantly influences suspension stability by increasing repulsive interactions between nanocellulose particles.[^160^, ^161^]

The application of TEMPO CNF as a template for the controlled release of antimicrobial copper from polyvinyl alcohol (PVA) films, demonstrating potential applications in antimicrobial coatings and packaging materials.^[162]^ These findings collectively underscore TEMPO‐mediated oxidation as a promising method with broad applications, showcasing improved properties and versatility in the biomedical and materials science domains.

##### 3.3.2.3 Bleaching

The optimization of bleaching methods has become crucial for maximizing cellulose extraction from various waste materials.^[163]^ In biomedical applications, bleaching involving treatment with sodium chlorite in an acid medium led to the removal of lignin through complex formation and depolymerization, resulting in a diameter decrease in fibrils to approximately 10–20 μm.[^164^, ^165^, ^166^]

In the context of utilizing agricultural residues, such as sago fronds, an underexploited lignocellulose waste, investigations into alkaline delignification and diverse bleaching agents have been conducted.^[167]^ Proximate analysis of sago fronds revealed their potential as a cellulose source, emphasizing the need for tailored bleaching conditions. Optimized parameters, such as 10 % NaOH for 2 hours followed by alkaline hydrogen peroxide, produced cellulose with elevated crystallinity (40.65 %) and whiteness, aligning with the global pursuit of sustainable materials for applications in eco‐friendly papermaking and composite materials.^[167]^

The study of sorghum straw and rice straw for cellulose production highlights a global initiative to make use of various agricultural by‐products. By optimizing pulping and bleaching processes—taking into account factors like cooking time, dilute alkali concentration, and bleach volume–research has produced pulps with low Kappa numbers, reduced lignin content, and cellulose with increased crystallinity. These optimized methods hold international importance due to their potential to drive the development of sustainable cellulose‐based materials, which could benefit industries ranging from textiles to cutting‐edge biomedical applications. Regarding concentration, the concentration of dilute alkali used in the pulping process significantly impacts the cellulose yield and quality. A higher alkali concentration can help in the effective removal of lignin, improving the purity of the cellulose. However, this must be balanced, as too high a concentration could lead to cellulose degradation or excessive energy consumption. The concentration of bleaching agents also influences the final quality of the cellulose, affecting both its crystallinity and the extent of lignin removal.^[168]^

Moreover, the extraction of CNC from rice straw using green bleaching processes with UV‐assisted oxidation of H_2_O_2_ provides a glimpse into the worldwide quest for eco‐friendly alternatives. The resulting CNC exhibited exceptional properties, including a crystalline index of 83.8 % and a high degradation temperature of 257 °C.^[169]^ Collectively, these global endeavors underscore the pivotal role of optimized bleaching methods in unlocking the full potential of cellulose from diverse sources, fostering advancements in sustainable material science.

##### 3.3.2.4 Mercerization

Mercerization, an effective treatment method, involves exposing nanocellulose to strong alkali solutions like NaOH.[^170^, ^171^, ^172^, ^173^] This process into CNC with allomorph II (CNC‐II) from mercerized cellulose revealed temperature as the most influential factor, resulting in CNC‐II with 80.89 % crystallinity and 5.21 nm crystallite size.^[174]^

The modified nanocellulose demonstrates enhanced properties for biomedical applications, as evidenced by improved chitosan composite strength with mercerized nanocellulose.[^175^, ^176^] Additionally, the mercerization treatment of bamboo fiber composites using a 6 % alkaline solution concentration led to significant improvements, including a 7 % increase in flexural strength, a 10 % rise in tensile strength, and an impressive 81 % boost in compressive strength.^[177]^ Moreover, the alkali treatment resulted in a notable reduction in water absorption, decreasing from 51 % to 35 % after a 30 minute exposure to a 5 % NaOH solution at room temperature.^[178]^

##### 3.3.2.5 Esterification

Esterification is a versatile modification technique to tailor nanocellulose properties for biomedical applications. Esterification can attach new functional groups, improve stability and solubility, and add antimicrobial or sensing abilities to nanocellulose. Controlling reaction conditions like the catalyst type enables effective esterification for the desired modifications.[^179^, ^180^] Studies have utilized various esterification reactions like acylation, carboxymethylation, and succinylation to add new functional groups to nanocellulose.^[181]^ These modifications make nanocellulose more hydrophobic, thermally stable, and compatible with other polymers. Reaction conditions like temperature, time, and the type of catalyst impact the degree of esterification. Alkaline salt catalysts improved the esterification of cellulose by up to 95 % compared to no catalyst.^[182]^ Ionic liquid/molecular solvent systems increased the esterification rate by 2–3 times and selectivity by 15–20 % over molecular solvents alone.^[183]^ Lipase catalysts also acetylated nanocellulose effectively, with a degree of substitution ranging from 0.1 to 2.9.^[184]^

##### 3.3.2.6 Acetylation

Acetylation of nanocellulose is the most widely studied esterification reaction. Acetylation is a treatment technique that can alter the properties of nanocellulose, making it more suitable for biomedical applications. Studies have shown that acetylation makes nanocellulose more hydrophobic and thermally stable by replacing surface hydroxyl groups with acetyl groups.[^185^, ^186^, ^187^] The increased hydrophobicity improves solubility and reduces water interactions. Acetylation also increases the flexibility of the nanocellulose surface while decreasing crystallinity.[^185^, ^188^]

Acetylation beneficially alters nanocellulose properties like hydrophobicity, solubility, and thermal stability. The improved characteristics make acetylated nanocellulose suitable for applications like biomedical devices. Acetylation is thus a useful modification technique for tailoring nanocellulose to meet performance requirements in biomedical and other applications.[^189^, ^190^] Higher acetylation and mannose content improved solubility and interaction with cellulose. Acetylated nanocellulose had better thermal stability, mechanical properties, and water resistance compared to unmodified nanocellulose.^[191]^

##### 3.3.2.7 Etherification

Etherification involves the reaction between the hydroxyl groups on the nanocellulose surface and ether‐forming reagents such as alkyl halides or epoxides. This reaction results in the introduction of ether groups on the nanocellulose surface, which improves its hydrophobicity and dispersibility in organic solvents. The resulting etherified nanocellulose can be used for the production of various nanocomposites and coatings to introduce ether groups.^[187]^ This increases hydrophobicity and dispersibility in organic solvents.^[192]^ The increased dispersibility of nanocellulose in PLA through etherification is influenced by the degree of substitution, with higher substitution enhancing the compatibility of nanocellulose with the polymer.^[193]^

Various characterization techniques like Nuclear magnetic resonance (NMR) spectroscopy, scanning electron microscope (SEM), and zeta potential analysis are used to confirm etherification and resulting property changes.[^194^, ^195^] The sono‐assisted etherification process's successful conversion of maize stalk pith into cationic CNP with favorable properties opens avenues for applications in the medical industry. These highly charged and well‐dispersed CNP, with unique dot‐like shapes and a positive zeta potential of up to +40 mV, can be explored for drug delivery systems, wound healing materials, and tissue engineering.^[196]^ The sustainable utilization of agricultural residues in producing functional nanomaterials aligns with eco‐friendly practices, offering potential innovations in medical applications.^[197]^

##### 3.3.2.8 Hydrolysis with Metal Salt Catalysts

Various catalysts, including transition metal salts, are essential for improving the efficiency of cellulose hydrolysis. Transition metal salts are classified into monovalent (*e. g*., NaCl, KCl), divalent (*e. g*., CaCl_2_, FeCl_2_, FeSO_4_, Mn(NO_3_)_2_), and trivalent (*e. g*., FeCl_3_, Fe_2_(SO_4_)_3_, Al(NO_3_)_3_, Cr(NO_3_)_3_) categories based on their valence states.[^198^, ^199^] The valence state of the catalyst influences the hydrolysis process by polarizing water molecules and producing hydronium ions, which act as co‐catalysts, thereby enhancing the efficiency of the reaction.^[200]^

Studies reveal the impact of specific transition metal salts on cellulose properties. For instance, the addition of Fe(III) ions significantly improved nanocellulose crystallinity by 19 % compared to native cellulose.[^201^, ^202^] Similarly, Ni(II) inorganic salt increased nanocellulose crystallinity, emphasizing the role of transition metals in influencing cellulose structure.^[203]^ In the preparation of CNC the efficiency of Fe(NO_3_)_3_, Co(NO_3_)_2_, and Ni(NO_3_)_2_ as co‐catalysts with H_2_SO_4_ demonstrated selective degradation of cellulose's amorphous structure, resulting in larger crystallites and higher crystallinity indices compared to native cellulose.^[204]^

The choice of transition metal salt is crucial, as it impacts the final product's yield and properties. The trivalent oxidation state of Fe(III) cations, for instance, led to more effective hydrolysis and smaller CNCs compared to divalent cations (Co(II) and Ni(II)). Tailoring the catalyst selection based on specific hydrolysis requirements ensures the desired product properties and process efficiency. For instance, catalysis with nickel resulted in shorter and lower aspect ratio CNCs compared to acid hydrolysis‐produced nanocellulose.^[203]^ The careful consideration of catalysts is essential for optimizing cellulose hydrolysis outcomes.

##### 3.3.2.9 Hydrolysis with Liquid and Gaseous Acids

The application of acid hydrolysis for CNC preparation is a common method but is associated with drawbacks such as poor thermal stability, equipment corrosion, and environmental concerns. Replacing liquid acids with solid acids, which offer advantages such as better thermal stability of the resulting CNC and easy recovery and recycling of the solid acid.^[205]^ Concentrated phosphotungstic acid (H_3_PW12O_4_0) hydrolysis is used to prepare rod‐like CNC with good thermal stability. However, the high cost of solid acid catalysts and the extended hydrolysis time are significant drawbacks of this method.^[206]^

To overcome these limitations, solid phosphotungstic acid hydrolysis with a sonication approach significantly reduced the operating time from 30 hours to 10 minutes while maintaining high CNC crystallinity and yield.^[207]^ The data indicated a remarkable enhancement in efficiency.

Gaseous acid hydrolysis presents an alternative technique, involving hydrolyzing wet cellulose with acidic gas. This process emphasizes the need for additional mechanical treatments like grinding or ultrasonication for further CNC production.^[208]^ The data suggested that this method could offer advantages with proper post‐treatment steps.

##### 3.3.2.10 Periodate Oxidation

Periodate oxidation method involves selective cleavage of the C2−C3 bonds in cellulose using sodium periodate to generate aldehyde groups on the nanocellulose surface. The aldehyde groups can then undergo further reactions with amines, hydrazines, or other nucleophiles to introduce desired functionalities.[^209^, ^210^]

Studies have highlighted the efficacy of periodate oxidation in enhancing nanocellulose properties for composites, coatings, and biomedical applications. The oxidation improves dispersibility in aqueous systems and reactivity towards chemical modifications.^[211]^ It also facilitates crosslinking with polymers like chitosan to form nanocomposites, while allowing nanoparticle templating on the nanocellulose surface.^[212]^ Furthermore, the method generates carboxylated CNC that act as effective reinforcing agents in PVA films.^[213]^

Periodate oxidation also shows potential for tailoring nanocellulose for biomedical uses. Conversion to carboxylated CNC introduces surface charges that improve colloidal stability,^[214]^ preventing aggregation in physiological conditions. The aldehyde groups generated can also conjugate bioactive molecules for controlled drug delivery.[^180^, ^186^]

##### 3.3.2.11 Reduction

The reduction chemical method is commonly employed for nanocellulose surface modification, involving the use of reducing agents like sodium borohydride (NaBH_4_) to reduce functional groups on the nanocellulose surface.^[215]^ This allows further functionalization with hydrophobic molecules to create a hydrophobic surface coating. Studies have shown the efficacy of this method in modifying CNF to improve dispersibility in hydrophobic polymer matrices like PVA.^[216]^ The enhanced compatibility resulted in improved mechanical properties of CNF‐reinforced composites, with increases in tensile strength.

Additionally, combining reduction with other surface treatments can further augment nanocellulose properties. For example, reduction followed by esterification with alkyl ketene dimer was found to improve hydrophobicity and polyethylene matrix compatibility. This resulted in a 70 % increase in elongation at break for the composites with modified cellulose versus unmodified.^[217]^ While concerns exist regarding the toxicity of reducing agents like NaBH_4_, the reduction method remains promising for tailoring nanocellulose compatibility and filler reinforcement capacity in composites for biomedical applications like bone repair scaffolds and tissue engineering constructs.[^218^, ^219^]

##### 3.3.2.12 Silanization

Silanization is an effective surface modification technique for improving nanocellulose properties like hydrophobicity, thermal stability, and mechanical strength.^[220]^ Silanization through solution and vapor techniques effectively modifies nanocellulose for biomedical applications.^[221]^ The process improves mechanical, thermal, and surface properties while also adding antimicrobial, biocompatible, or oil repellent functions. The process entails covalently bonding silane molecules to nanocellulose, achieved through either solution‐based or vapor‐phase methods. Notably, solution methods, with just 0.10 wt % of the surface‐modified BNC added, result in a substantial increase in tensile strength and Young's modulus by factors of 1.6 and 1.8, respectively, without compromising the maximum elongation rate.^[222]^ Vapor methods reduce viscosity by crosslinking surface silane networks, at equivalent concentrations.^[223]^

Silanization also adds beneficial functionality to nanocellulose. Aminosilanes impart non‐leaching antimicrobial activity against gram positive and negative bacteria.^[224]^ Carboxylsilanes promote cell viability and proliferation, improving nanocellulose biocompatibility for tissue scaffolds.^[225]^ Alkylsilanes produced oleophobic nanocellulose aerogels with tunable surface wetting and oil repellency.^[226]^

##### 3.3.2.13 Grafting

Grafting treatments, in particular ring‐opening polymerization (ROP) and surface‐initiated atom transfer radical polymerization (SI‐ATRP), have become pivotal strategies for tailoring nanocellulose surfaces with diverse polymers to impart enhanced properties and functionalities.^[227]^

ROP, commonly facilitated by stannous octoate (Sn(Oct)_2_) as a catalyst, enables the hydroxyl groups on nanocellulose to initiate ring‐opening processes. This results in high‐density polymer grafts with fine‐tuned chain lengths while preserving the nanocellulose structure. ROP has been widely explored for modifying nanocellulose with polymers like poly (ϵ‐caprolactone) (PCL), leading to notable improvements in mechanical strength (over 20 % increase) and interfacial adhesion (15–30 % increase) between nanocellulose and polymer matrices.[^228^, ^229^, ^230^] Studies have optimized ROP conditions including catalyst type and amount, polymerization time, and use of sacrificial initiators to control graft density and chain length.

In parallel, SI‐ATRP has become prominent for synthesizing well‐defined polymer brushes on nanocellulose with high grafting density, controlled chain length, and low polydispersity. In this copper‐mediated approach, nanocellulose is first functionalized with a brominated initiator, followed by polymerization of desired monomers from the activated surface sites. SI‐ATRP has enabled the grafting of various responsive polymers from nanocellulose to create stimuli‐responsive platforms. For instance, poly (N‐vinylcaprolactam) and poly (oligoethylene glycol) methyl ether acrylate grafted nanocellulose exhibit reversible aggregation in response to temperature changes.[^231^, ^232^] Dual functional systems responding to both temperature and pH have also been synthesized using SI‐ATRP.^[233]^

While powerful, SI‐ATRP grafting has to be carefully controlled to minimize unintended nanocellulose backbone degradation and side reactions.^[234]^ Grafting polymer chain length and density greatly influence resulting properties. In general, long grafts enhance matrix‐filler interactions but may reduce colloidal stability. Shorter grafts preserve nanocellulose morphology while still improving dispersion. ROP and SI‐ATRP have become indispensable tools for functionalizing nanocellulose through surface‐initiated polymerizations, enabling substantial property improvements. The techniques provide unparalleled control over grafting parameters to tailor nanocellulose with advanced responsive behaviors for diverse cutting‐edge applications.^[188]^

##### 3.3.2.14 Electrochemical Processes/ Plasma

Plasma treatment is an effective surface modification technique for altering the properties of cellulose‐based materials. Plasmas contain reactive species that can selectively functionalize surfaces with oxygen, nitrogen, or other functional groups. An atmospheric pressure dielectric barrier discharge plasma was employed to polymerize styrene onto cotton fibers, conferring new hydrophobic properties as confirmed by SEM and FTIR analysis.^[235]^ Cellulose fibers treated with oxygen plasma enable uniform deposition of ZnO nanoparticles, achieving UV‐blocking ability.^[236]^ The combined plasma etching of cotton fabrics with TiO_2_ nanoparticle coating to achieve enhanced UV protection.^[237]^

In addition to UV and hydrophobic functionality, plasma treatment can improve other cellulose material properties such as thermal insulation capacity and oil absorption for environmental remediation. The application of air plasma on freeze‐dried cellulose aerogels demonstrated a reduction in thermal conductivity by over 50 %.^[238]^ While paper‐based cellulose aerogels were made superhydrophobic using plasma, the treatment led to boosting the aerogel's oil absorption capacity to support a load over 300 times its own weight.^[239]^ Atmospheric plasma enabled smoother and denser nanocellulose coatings on polyester and nylon compared to laser treatment.^[240]^

Further research points towards the potential of plasma treatment in functionalizing CNC and aerogels, including grafting surface groups and initiating polymerization reactions.^[241]^ The adaptability and superior effects achievable through plasma treatment position it as a valuable tool for tailoring cellulose materials for next‐generation functional devices and biomedical technologies.^[242]^ Integrating plasma processing with nanocellulose production opens avenues for renewable platforms with highly tunable architectures and chemistries, showcasing the potential for practical applications.

Studies have demonstrated the improved water resistance and mechanical strength of cellulose films for food packaging through ascent cold plasma treatment and polymerization.^[243]^ Submerged liquid plasma was utilized for the surface functionalization of nanocellulose, resulting in improved mechanical and thermal properties.^[244]^ Plasma surface modification was employed on CNC to reinforce ABS.^[245]^ Additionally, enhanced water resistance and improved thermal stability in regenerated cellulose films were achieved through low‐pressure plasma treatment.^[241]^ Studies showcase atmospheric pressure plasma treatment's prowess in improving thermal stability and hydrophobicity of CNF, nanocrystals, and aerogels, respectively.[^246^, ^247^, ^248^]

Table 4 summarizes key findings for chemical treatment methods used in nanocellulose production, highlighting their impacts on properties such as thermal stability, mechanical strength, and dispersion.

**Table 4:** Summary of chemical Treatment Methods for Nanocellulose Production: Key Findings and Contributions.

Treatment Method	Key Findings
Acid Hydrolysis	– Effective for producing CNC from cellulose by removing amorphous regions. – Sulfate groups from H_2_SO_4_ impact thermal stability and dispersion. – H_3_PO_4_ hydrolysis offers better thermal stability and flame resistance compared to H_2_SO_4_
TEMPO‐Mediated Oxidation	– Introduces carboxylic acid groups, improving water dispersion and reactivity. – Enhances mechanical properties of CNF composites. – Effective in biomedical applications due to improved stability and functionality.
Bleaching	– Optimized bleaching parameters improve cellulose crystallinity and material properties. – Green bleaching processes show high crystalline index and thermal stability.
Mercerization	– Increases CNC crystallinity and improves mechanical properties in composites. – Enhances water absorption and reduces water uptake in composites. – Improves chitosan composite strength and flexibility.
Esterification	– Modifies nanocellulose to improve stability, solubility, and hydrophobicity. – Reaction conditions like catalyst type and temperature affect the degree of esterification. – Enhanced esterification rates achieved using ionic liquids and lipase catalysts.
Acetylation	– Replaces hydroxyl groups with acetyl groups, increasing hydrophobicity and thermal stability. – Improves flexibility while reducing crystallinity. – Enhances solubility and mechanical properties for biomedical applications.
Etherification	– Enhances compatibility with polymers like PLA. – Effective characterization techniques confirm successful etherification and property changes.
Hydrolysis with Metal Salt Catalysts	– Transition metal salts enhance cellulose hydrolysis efficiency and crystallinity. – Trivalent salts like Fe(III) offer more effective hydrolysis compared to divalent salts.
Hydrolysis with Liquid and Gaseous Acids	– Solid acids improve thermal stability of CNC but are costly and time‐consuming. – Gaseous acid hydrolysis requires additional mechanical treatments. – Solid phosphotungstic acid and sonication significantly reduce processing time while maintaining high CNC quality.
Periodate Oxidation	– Enhances dispersion and reactivity in composites and coatings. – Carboxylated CNC improves stability and enables controlled drug delivery.
Reduction	– Modifies surface to improve hydrophobicity and polymer matrix compatibility. – Effective for enhancing mechanical properties of CNF‐reinforced composites.
Silanization	– Solution and vapor methods improve tensile strength and Young's modulus. – Introduces antimicrobial and biocompatible properties for biomedical applications.
Grafting	– Ring‐opening polymerization (ROP) and surface‐initiated atom transfer radical polymerization (SI‐ATRP) modify surfaces for improved properties; allows for responsive behaviors.
Plasma Treatment	– Alters surface properties with reactive species; improves UV‐blocking, thermal insulation, and oil absorption. – Plasma treatment can functionalize nanocellulose for diverse applications, including environmental and biomedical uses.

#### 3.3.3 Biological Processes

Utilizing biological agents, such as enzymes, for the surface modification of nanocellulose constitutes biological methods. These methods encompass enzymatic treatment, genetic modification and bacterial surface display.^[249]^ Enzymatic treatment employs enzymes like cellulases to enhance the surface characteristics of nanocellulose, leading to improved solubility and reactivity.^[250]^ Genetic modification entails altering the genes of the organism responsible for nanocellulose production to enhance its properties.^[251]^ Bacterial surface display is a technique involving the presentation of proteins on the surface of bacteria.^[252]^

##### 3.3.3.1 Enzymatic Hydrolysis

Enzymatic hydrolysis involves utilizing cellulase enzymes to break down cellulose into glucose and nanocellulose crystals through the cleavage of glycosidic bonds. It is a highly tunable and sustainable nanocellulose production technique that may enable superior control over surface functionalities compared to conventional methods when reaction conditions and substrates are optimized. In a one‐step ball milling mechano‐enzymatic process, mechanical shearing forces and enzymatic activity synergistically combine, resulting in a 49.3 % CNC yield. This method not only reduces water usage but also follows first‐order reaction kinetics, dependent on rotation speed.^[253]^ Additionally, controlling enzymatic hydrolysis parameters such as temperature, pH, substrate concentration, and enzyme concentration enables tailored nanocellulose functionalization with specific surface groups.^[250]^

Enzymatic hydrolysis offers several advantages over the harsh acids and high temperatures used in conventional nanocellulose production. It allows for superior ethanol yields through simultaneous enzymatic saccharification and fermentation by optimizing factors such as enzyme loading, pH, and temperature.^[254]^ Furthermore, enzymatic hydrolysis produces nanocelluloses with more uniform size distributions and specific functionalities like carboxyl and hydroxyl groups, making them suitable for applications in drug delivery and tissue engineering. Additionally, enzymes provide high selectivity, efficiency, and operate under mild conditions.^[186]^

However, enzymes can be inhibited by structural factors and may require preprocessing to improve accessibility. In a hybrid enzymatic and acid hydrolysis process, it was found that enzymatic pretreatment enhanced nanocellulose crystallinity from softwood pulp to approximately 70 % by partially deconstructing amorphous regions while maintaining structural integrity for further sulfuric acid isolation.^[15]^

##### 3.3.3.2 Genetic Modification

Genetic modification involves introducing new DNA into an organism to alter its properties. By programming the biosynthetic machinery itself, rather than relying solely on post‐production processing, genetically engineered microbes provide unique opportunities for scalable bottom‐up fabrication of designer nanocelluloses for applications ranging from flexible electronics to tissue scaffolds.^[255]^ In the realm of cellulose production, this approach allows for tuning the metabolic pathways of cellulose‐synthesizing bacteria and fungi to optimize yield and tailor surface chemistry. Common strategies include overexpressing native or foreign genes that code for enzymes like beta‐glucosidase (BGL) using plasmids and strong promoters. BGL hydrolyzes cellobiose into glucose, overcoming rate limitations and inhibition. The expression of BGL in hosts enhances cellulase efficiency and economizes biomass saccharification.[^256^, ^257^]

Another avenue involves modifying cellulose synthase and complementary pathways to generate nanocelluloses with unique dimensions and crystallinities. For instance, researchers created *hypermotile Komagataeibacter hansenii* cells that produced thicker, loosely networked BNC ribbons.^[255]^ The resulting mutated BNC scaffolds demonstrated superior cellular adhesion for cartilage tissue engineering. There are also instances of genetic alterations contributing to the bio‐synthesis of curdlan/cellulose bio nanocomposites with genetically modified *gluconacetobacter* strains, resulting in materials with markedly enhanced mechanical strength.[^251^, ^258^]

In addition to an augmented quantity, these studies show that genetic engineering enables precise control over cellulose surface chemistry and morphology, which is unachievable with physical or chemical treatments. Custom proteins and graphene‐binding peptides self‐assemble on nanocelluloses, forming hierarchical biomimetic structures. Furthermore, the introduction of functional groups improves subsequent chemical modification efficiency, expands biochemical sensing capabilities, and tailors degradation.^[259]^

##### 3.3.3.3 Bacterial Surface Display

Bacterial surface display involves expressing target proteins fused to anchoring motifs on the exterior of bacterial cell walls. As a scaffold material, bacterial cellulose possesses remarkable biocompatibility, porous microstructure, and mechanical properties resembling human tissues.^[225]^ However, properties like conductivity, magnetic response, and bio‐resistance require augmentation for advanced applications. Bacterial surface display enables such functionalization by decorating cell surfaces with designed peptides that self‐assemble nanomaterials in situ during cellulose synthesis.^[260]^ Surface display is employed to bind graphene sheets to Gluconacetobacter cells, facilitating subsequent pyrolysis into lightweight, highly conductive carbonized bacterial cellulose foams for energy storage.^[261]^ Magnetic nanoparticles are applied to cellulose‐producing bacteria, resulting in magnetic nanocomposites with expanded bioseparation and imaging utility.^[262]^ Surface display systems are utilized for showcasing growth factors on bacterial cellulose, contributing to wound healing and tissue repair. Additionally, drug loading has been enhanced by employing conjugated small molecules on engineered cellulose‐producing cells.^[186]^ Beyond materials development, bacterial cellulose has direct applications as a food additive and packaging biomaterial.^[263]^ It's high‐water retention and 3D nanostructure provide novel rheological, textural, and stabilizing functionality. Bacterial surface engineering has produced cellulose‐based interfaces with anti‐biofouling capabilities for biosensing and filtration uses.^[264]^ Table 5 provides essential insights into biological treatment methods for producing nanocellulose.

**Table 5:** Summary of Biological Treatment Methods for Nanocellulose Production: Key Findings and Contributions.

Treatment Method	Key Findings
Enzymatic Hydrolysis	– Utilizes cellulase enzymes to convert cellulose into nanocellulose with enhanced surface functionalities, leading to improved solubility and reactivity. – Offers precise control over size and surface properties by optimizing parameters like temperature and enzyme concentration. – Results in high ethanol yields and uniform nanocellulose size distributions with applications in drug delivery and tissue engineering.
Genetic Modification	– Introduces new DNA into microorganisms to enhance cellulose production and tailor nanocellulose properties, such as surface chemistry and crystallinity. – Facilitates precise control over cellulose synthesis and surface modification, improving material strength and bioactivity.
Bacterial Surface Display	– Involves expressing target proteins on bacterial cell surfaces to functionalize nanocellulose with unique properties, such as conductivity and magnetic response. – Allows the creation of advanced materials like conductive foams and magnetic nanocomposites for energy storage and imaging. – Enhances bacterial cellulose for applications in wound healing, drug delivery, and food packaging.

### 3.4 Post Treatment

Post‐treatment processes in nanocellulose production refer to additional steps or modifications applied to nanocellulose after the initial extraction or synthesis to enhance specific properties or functionalities. These processes are designed to tailor the nanocellulose material for particular applications. Several common post‐treatment processes in nanocellulose production include surface coating, crosslinking, sulfation, ionic liquids treatment, and drying.

Surface coating: In the context of nanocellulose production for biomedical applications, post‐treatment processes, particularly the application of coatings, have emerged as pivotal factors. A novel method employing elastically bonded grinding wheels for post‐treatment of coated cutting tools has been proposed, focusing on enhancing the removal rate of surface inhomogeneities and fortifying coating adhesion strength.^[265]^ Additionally, carbide‐based thermal spray coatings have shown promise in biomedical applications due to their resistance to degradation in harsh environments, with the composition, grain size, and post‐processing techniques influencing performance.^[266]^

Micro‐abrasive blasting, implemented as a treatment process on Tungsten carbide‐cobalt (WC−Co) substrates, has demonstrated a significant impact on improving the adhesion and tribological properties of TiN hard coatings, presenting potential applications in biomedical devices.^[267]^ Studies on mechanical surface treatment methods, including micro‐blasting, polishing, and buffing, along with plasma nitriding, have revealed their efficacy in improving tribological properties and adhesion of coating systems, making them valuable considerations for biomedical applications.^[268]^

Post‐treatment processes, such as laser and electron beam treatment, have showcased their effectiveness in augmenting the wear and corrosion resistance of thermal spray coatings on magnesium substrates, offering possibilities for biomedical implant materials.^[269]^ Furthermore, a comprehensive analysis of WC−Co substrate surface pre‐treatment and physical vapour deposition (PVD) coating post‐treatment has highlighted their significant influence on surface characteristics, minimizing the running‐in period, and reducing the coefficient of friction (CoF) in dry sliding applications–crucial considerations for biomedical applications.^[268]^

#### 3.4.1 Crosslinking

Crosslinking involves the formation of covalent bonds between nanocellulose chains, enhancing its mechanical strength and stability. Crosslinked nanocellulose is often used in applications requiring higher durability, such as tissue engineering scaffolds and drug delivery. Several common methods for crosslinking include carbodiimide coupling, esterification, polycarboxylic acids, and photochemical reactions.^[270]^ The utilization of carbodiimide chemistry, specifically employing 1‐ethyl‐3‐(3‐dimethylaminopropyl) carbodiimide (EDC) and N‐hydroxysuccinimide (NHS), allows for the activation of carboxyl groups present on nanocellulose, thereby facilitating the formation of covalent bonds with amines found on biomolecules.^[180]^ To illustrate, the crosslinking of TEMPO‐oxidized CNF (TCNF) with gelatin using EDC/NHS results in the creation of stable and supramolecular hydrogels possessing a storage modulus, rendering them suitable for use as bioinks.^[271]^ Polycarboxylic acids, such as citric acid, have the ability to directly react with cellulose hydroxyls through etherification or esterification, without necessitating the activation of carbodiimides.^[272]^

#### 3.4.2 Sulfation

Introducing sulfate groups to nanocellulose can enhance its water solubility, making it suitable for specific applications like drug delivery or biomedical coatings. Sulfation emerges as a versatile post‐treatment process in nanocellulose production, demonstrating its potential for various applications. In a study employing a reactive deep eutectic solvent (DES), wood cellulose pulp was sulfated, achieving an anionic charge of 3 mmol/g with a degree of substitution of 0.68. The sulfated cellulose nanofibers (SCNFs) exhibited a width of approximately 4 nm, making them promising candidates for applications as rheology modifiers or reinforcing additives in biomedical contexts.^[273]^ While sulfation reactions, particularly with sulfuric acid hydrolysis, have traditionally been employed for CNC production, challenges arise due to poor thermal stability. However, sulfonated nanocellulose finds applications in water purification for heavy metal removal, benefiting from soft‐soft interactions, and offers good pH stability in the presence of multivalent ligands.[^274^, ^275^] The use of mild sulfating agents like sulfamic acid, forming reactive DES, addresses some limitations associated with traditional methods. The resulting sulfated cellulose products show promise in terms of sulfate content and solution stability, offering advantages over sulfuric acid‐based processes. The sulfation of *Posidonia oceanica* waste for cellulose micro/nanofibril production indicates the potential of sulfonated pulps in obtaining high‐quality nanocomposites with reduced energy consumption. Sulfamic acid, employed in DES, proves to be a mild and effective sulfating agent, offering advantages in terms of toxicity and handling compared to traditional reagents like sulfuric acid.^[114]^

#### 3.4.3 Ionic Liquids Treatment

The production of nanocellulose using hydrolysis with ionic liquids, such as 1‐ethyl‐3‐methylimidazole acetate (EmimOAc) and 1‐allyl‐3‐methylimidazolium chloride (AmimCl), from enzymatically pre‐treated microcrystalline celluloses (Avicel and Whatman) has shown promising results.[^276^, ^277^] The nanocellulose, analyzed by FTIR, XRD, DLS, SEM, and TG, has a regular, spherical structure with diameters of 30–40 nm.^[80]^ While it shows lower crystallinity and thermal stability compared to materials produced via Trichoderma reesei enzyme hydrolysis, the two‐step enzymatic treatment and ionic liquid hydrolysis method is effective. Ionic liquids, as green solvents, offer benefits like stability, low melting points, and recyclability, but challenges like high costs and extraction inefficiency remain.^[278]^ In addressing environmental concerns, researchers increasingly turn to ionic liquids as recyclable mediums for nanocellulose processing, emphasizing the diverse applications and properties of nanocellulose extracted by different ionic liquids.^[278]^ Additionally, Haron's review underscores the pivotal role of ionic liquids in addressing challenges related to the petrochemical industry and environmental pollution. Ionic liquids are portrayed as green solvents enabling the efficient isolation and modification of nanocellulose under mild conditions. The review explores the impact of different ionic liquid cations/anions on the structural changes of nanocellulose, emphasizing their role in surface modification reactions and promoting sustainable commercialization through adherence to green chemistry principles and ongoing research and development efforts.^[279]^

#### 3.4.4 Drying

Efficient drying methods for nanocellulose without inducing aggregation have been a crucial focus for researchers. Various drying techniques include spray drying, freeze drying, and supercritical drying. However, challenges persist due to issues such as fiber agglomeration and the need for expensive equipment. By comparing drying methods, it is evident that spray drying produces nanocellulose with desirable nano and micron‐sized fibers. Friable aggregates with excellent biodegradability are effectively produced by freeze drying.^[280]^ Despite its potential, the industrial utility of freeze drying is limited by the high cost of liquid nitrogen. Treatment with water‐soluble polymers is hindered by impracticality and expense, restricting industrial application.[^281^, ^282^] The negative impact on mechanical strength has been revealed through surfactant treatment to improve fiber dispersion.^[283]^ Enhancement in dewatering and reduction in water retention during drying has shown promise with the cationic surfactant CTAB.^[284]^ Furthermore, the challenges of oven‐drying have been addressed by adding *tertbutanol*, significantly reducing drying times and maintaining excellent dispersibility.^[285]^ Despite these advancements, the choice of the drying method significantly influences nanocellulose properties, emphasizing the need for tailored approaches based on specific applications. Overall, the quest for cost‐effective, scalable, and environmentally friendly drying processes remains vital for the successful commercialization of nanocellulose.

The choice of post‐treatment processes depends on the intended applications of nanocellulose and the desired properties for the final product. Researchers and manufacturers often explore various post‐treatment strategies to unlock the full potential of nanocellulose in diverse fields. Table 6 summarizes key findings about post‐treatment processes in nanocellulose production.

**Table 6:** Summary of post‐treatment processes for Nanocellulose Production: Key Findings and Contributions.

Treatment Method	Key Findings
Surface Coating	– Enhanced removal rate and coating adhesion strength with elastically bonded grinding wheels. – Improved tribological properties with micro‐abrasive blasting. – Laser and electron beam treatments improve wear and corrosion resistance.
Crosslinking	– Carbodiimide coupling (EDC/NHS) used for stable hydrogels with improved storage modulus. – Polycarboxylic acids directly react with cellulose hydroxyls.
Sulfation	– Sulfated cellulose nanofibers (SCNFs) show potential for rheology modification and biomedical applications. – Sulfamic acid provides mild, effective sulfation with advantages over sulfuric acid.
Ionic Liquids Treatment	– Ionic liquids like EmimOAc and AmimCl yield spherical nanocellulose with unique properties. – Challenges include high costs and lower extraction efficiency.
Drying	– Spray drying produces desirable nano and micron‐sized fibers. – Freeze drying yields friable aggregates but is costly. – Tertbutanol addition improves oven‐drying efficiency.

Table 7 offers a comprehensive overview of key parameters critical to understanding the properties and characteristics of nanocellulose derived from waste materials. It includes purification techniques, bleaching methods, pre‐treatment processes, type of nanocellulose, crystallinity, thermal stability, morphology, and yield. The table highlights a diverse range of waste materials used in nanocellulose production. This diversity highlights the versatility of nanocellulose production and its potential to address waste management issues across various industries. By transforming waste into valuable nanomaterials, this approach embodies the principles of circular economy and sustainability. The wide range of source materials also suggests that nanocellulose production could be adapted to local waste streams, potentially reducing transportation costs and environmental impacts. The data reveals a variety of processing methods, often combining multiple techniques such as chemical treatments (acid hydrolysis, alkali treatment), mechanical processes (grinding, homogenization), and enzymatic treatments. The choice of method significantly influences the final properties of the nanocellulose. For instance, acid hydrolysis tends to produce CNCs with higher crystallinity, while mechanical methods often result in CNFs with longer lengths. The most effective approaches frequently involve a combination of methods, such as alkali pretreatment followed by acid hydrolysis or mechanical processing, indicating the complexity of optimizing nanocellulose production from waste materials.

**Table 7:** Characteristics and Properties of Nanocellulose from Waste Materials.

Waste Materials		Purification Methods	Bleaching Matrial	Pre‐ Treatment	Treatment	Type of Nanocellulose	Crystallinity (%)	Thermal Stability (°C)	Morphology	Yield
Textile Waste Material	Industrial cotton waste^[63]^	Sulfuric acid (95–98 %), sodium hydroxide pellets (97 %), hydrogen peroxide (35 % v/v) from Sigma‐Aldrich and Dinâmica.	Alkali, H_2_O_2_, NaOH, 1 : 1 ratio.	Acid Pre‐treatment	acid hydrolysis	nanocellulose	75–81	146–200	Length: 5880 nm, Width: 105 nm	53–83
	Bombyx mori silk^[286]^	Sulfuric acid	Sulfuric acid, H_2_O_2_, 3 : 1 ratio.		high‐pressure homogenization	nanofibers			Diameter: 2−4 μm, Width: 200–300 nm	
Paper Waste Material	Wood flour^[287]^	Alkaline extraction: 10 % wt . /v calcium hypochlorite (Synth, Brazil), 1 % v/v acetic acid, 1 : 10 ratio, 1 h, 70 °C.	Sulfuric acid, hydrogen peroxide, 3 : 1.	Enzymatic treatment	Enzymatic treatment	nano structures	60–80		Length: 100–200 nm, Width: 60–100 nm	2–12 %
	Seond paper waste^[288]^	Alkaline extraction: 5 % NaOH, 10 % H_2_O_2_, 10 % sodium silicate, 60 °C, 1.5 h.		chemical pretreatment with hydrogen peroxide and sodium hydroxide	acid hydrolysis	nanocellulose	66–74	lower than that of untreated cellulose	Diameter: 10–30 nm, Length: 300–600 nm	35
	Waste newspaper^[289]^	Acid hydrolysis: sulfuric acid (H_2_SO_4_), 20–60 wt . %.	NaClO_2_, 1.7 % w/v, pH 4.5, 125 °C, 4 hours	Alkali treatment (NaOH)	acid hydrolysis	Nanocellulose	76		Diameter: 3–10 nm, length: 100–300 nm^[22]^	47
	Waste board and milk container board^[290]^	Washing and screening with Somerville screen.		Alkali treatment (NaOH)	Grinding	Nanofibrillated cellulose	47–61		Diameter: 2–80 nm	
	Mixture of cardboard and newspaper wastes^[291]^	Ozonization (0.35 L/min) for 0–120 min at room temperature, followed by hydrolysis with 70 % maleic acid at 100 °C for 90 min.		Acid Pre‐treatment	Ultrasonication	CNC	71	300	Diameter: 165 nm, Length: 2431 nm	1
	Flax fibers^[292]^	Sodium hydroxide, bleaching, sodium chlorite, potassium hydroxide treatment.	Sodium hydroxide	Alkali treatment (NaOH)	ultrasonication and acid hydrolysis	cellulose nanowhiskers	–		Diameter: 10–16 nm, Length: 200–400 nm	–
Agricultural Waste Material	Sugarcane Bagasse^[293]^	Sodium hydroxide	1 % w/v NaOH, 1.5 hours, 90 °C.	ionic liquid	Homogenization	nanocellulose	52	238	Diameter: 10–20 nm	–
	Pineapple Leaf^[110]^	Sodium hydroxide, acetic acid, sodium hypochlorite, oxalic acid treatments.	NaOH, acetic acid.	steam explosion	steam explosion blending	nanocellulose	74	–	Width: 5–60 nm	–
	Wheat Straw^[294]^	Hydrogen peroxide treatment.		Acid Pre‐treatment	grinding	CNF				
	Corn Stover^[295]^	sodium hydroxide treatment, bleaching.		Alkali treatment (NaOH)	ultrasonication and acid hydrolysis	CNC	–	–	Diameter: 4 nm, Length: 200 nm	–
	Rice straw^[296]^	Toluene, ethanol, sodium chlorite, potassium hydroxide treatments.	NaClO_2_		TEMPO‐mediated oxidation	CNC	70–90	160–310	Length: 100–200 nm, Width: 1–8 nm	[5–20]
	Coconut husk fiber^[297]^	Sodium hydroxide, acid hydrolysis, bleaching with sodium chlorite, glacial acetic acid.	1.5 g NaClO_2_, 8–10 drops glacial acetic acid.	Alkali treatment (NaOH)	acid hydrolysis	cellulose nanowhiskers	50–65	200–500	Diameter: 1–5 nm	‐
	Kenaf hemp^[298]^	Sodium hydroxide, bleaching, anthraquinone treatment.	alkali and consecutive bleaching	Alkali treatment (NaOH)	grinding	CNF	82	200–400	Diameter: 1–30 nm	58
Wood Waste Material	Sawdust^[299]^	alkali treatment followed by high‐pressure homogenization	NaOH, CH_3_COOH, NaOCl, 10 % HCl, sonicated, 2 h, 40 °C.		Homogenization	CNC	66–80	300–350	Diameter: 18–35 nm, Length: 101–107 nm.	47–59
	Bamboo^[300]^	Mechanochemical approach with the dissolving action of phosphoric acid on cellulose	phosphoric acid		ultrasonication	CNC	60–67	240–350	Diameter: 15–30 nm, Length: 100–200 nm	77.37
	Jute^[301]^	alkali and Oxalic acid treatment followed by steam explosion	NaClO_2_ solution, pH 2.3, 1 h, 50 °C.	Steam explosion	alkali treatment	Nanocomposites	62–83	250–400	Diameter: 50 nm, Length: few mm	21–58
	Eucalyptus^[302]^	Mechanical (refining and sonication) and chemical (acid hydrolysis)	Hydrolysis, sulfuric acid, 60 % v/v, 45 °C.	–	acid hydrolysis	crystalline nanofibers	40–53	230	Diameter: 10–30 nm, Length: 1–50 mm	61–65
Animal Waste Material	Cow Dung^[68]^	Pre‐hydrolyzed filtrate treated with 3 ml of 72 % H_2_SO_4_ for 1 h at 30±0.5 °C, stirred occasionally on a water bath.	Hypochlorite, Hydrogen peroxide	Acid Pre‐treatment	acid hydrolysis	Nanocellulose	48–53		Length: 100–400 nm, Width: 30 nm	11
	Horse Dung^[303]^	Acid detergent fiber solution: 10 g acid detergent powder, 0.5 mL decahydronaphthalene in 500 mL 0.5 M sulfuric acid. Dried residue treated with 72 % H_2_SO_4_.	–	Acid Pre‐treatment	–	nanolignocellulosic	–	–	Length: 25 nm, Width: 2 nm	58–82
	Elephant Dung^[17]^	Extraction with organic solvents like hexane and dichloromethane, followed by multiple bleaching steps using NaClO_2_.	mechanical and enzymatical pretreatment	Alkali treatment (NaOH)	alkali treatment	Nanocellulose	–	–		[12–41]
Food waste	Tomato Peel^[304]^	Sodium hydroxide, bleaching, sodium chlorite.	4 % H_2_O_2_, pH 11.5, 45 °C, 6 h.	Alkali treatment (NaOH)	acid hydrolysis	CNC	70–80	275–357	Length: 135 nm, Width: 7 nm	[10–16]
	Banana^[113]^	Sodium hydroxide acetic acid.	NaOH, acetic acid (27 g, 78.8 g), and a 1 : 3 volume mixture of sodium hypochlorite solution.	Steam explosion	acid hydrolysis	CNF	–	370–380	Width: 5–30 nm	very high
	Soybean pods^[305]^	Sodium hydroxide, hydrochloric acid, chlorine dioxide treatments.	ClO_2_, pH 2.3, 1 h, 50 °C.	Alkali treatment (NaOH)	Homogenization	nanofibers			Diameter: 50–100 nm, Length: micrometer	50–60
	Orange waste[^306^, ^307^]	Enzymatic treatment, sodium hydroxide, sodium chlorite treatments.		enzymatic hydrolysis	Ultrasonication	nanocellulose	60		Length: 150–260 nm, Width: 1–1.5 nm	
	Oil palm biomass residue^[308]^	Acid hydrolysis	Oxygen, ozone, hydrogen peroxide.	–	acid hydrolysis	MCC	–	300–400	Length: 100–300 nm, Width: 3–10 nm	–
	Carrot residue^[309]^	Sodium hydroxide treatment, bleaching.	Aqueous chlorite (1.7 wt % in water) with distilled water, 80 °C, 4 h, under 4 times mechanical stirring.	enzymatic hydrolysis	grinding	nanofibers			Length: 200–300 nm, Width: 6–8 nm	

The properties of the produced nanocellulose vary widely, reflecting the diversity of source materials and processing methods. Crystallinity ranges from 40 % to over 80 %, with agricultural and wood wastes generally yielding higher crystallinity. Thermal stability typically falls between 200 °C and 400 °C, often showing improvement over untreated cellulose. Particle dimensions vary greatly, with diameters from 1 to 100 nm and lengths from 100 nm to several micrometers. This variability in properties suggests that nanocellulose from waste could be tailored for diverse applications, from reinforcing materials in composites to potential uses in high‐temperature processes.

The yield of nanocellulose from waste materials varies dramatically, ranging from as low as 1 % to as high as 83 %. This wide range reflects the significant impact of both the source material and the extraction method on production efficiency. Higher yields are generally associated with less aggressive treatments, but these may result in larger, less uniform particles. The variability in yield presents both a challenge and an opportunity for optimization in the production process, highlighting the need for further research to improve efficiency and consistency across different waste streams.

While the data demonstrates the feasibility of producing nanocellulose from waste, it also reveals challenges in consistency of yield and quality, likely due to the heterogeneous nature of waste feedstocks. However, these challenges also present opportunities for process optimization and standardization. The variety of properties achieved suggests potential for tailoring nanocellulose for specific applications, from high‐strength materials to thermally stable products. As research in this field progresses, we can expect refinement of processes leading to more consistent and customized nanocellulose products. The development of efficient, scalable methods for nanocellulose production from waste could significantly impact both waste management practices and the availability of advanced, sustainable materials for various industries.

## 4 Biomedical Applications

The integration of nanocellulose derived from waste materials into biomedical applications represents a significant advancement in sustainable healthcare solutions. This innovative approach not only addresses waste management challenges but also introduces novel materials with exceptional properties suitable for various medical applications. The distinctive characteristics of nanocellulose, including its mechanical strength, adjustable porosity, and inherent biodegradability, make it particularly valuable for biomedical applications.[^310^, ^311^] Its chemical stability and biocompatibility ensure safe integration within biological systems, while its natural decomposition prevents tissue damage.^[312]^

The versatility of waste‐derived nanocellulose spans multiple critical areas in healthcare, including wound healing, drug delivery, biosensing, 3D‐bioprinting, and cosmetic applications. In wound healing, nanocellulose's significant moisture retention capabilities and mechanical robustness make it particularly effective for advanced wound dressings.^[313]^ The material's ability to incorporate bioactive agents and biopolymers enhances healing processes while reducing infection risks, contributing to more sustainable healthcare practices.^[314]^

Drug delivery systems benefit from nanocellulose's extensive surface area and adjustable surface characteristics, enabling controlled release mechanisms and improved therapeutic efficacy.^[315]^ The material's adaptability allows for various drug delivery formats, from tablets to aerogels, offering flexible release times and diverse application methods.^[316]^

In biosensing applications, nanocellulose‐based platforms demonstrate remarkable potential for health monitoring and diagnostics, particularly in the development of portable wearable devices that combine convenience with minimal sample preparation requirements.^[317]^

The field of 3D‐bioprinting has particularly benefited from nanocellulose's unique properties. Its superior rheological characteristics and biocompatibility make it an excellent choice for bioinks used in tissue engineering applications.^[318]^ When combined with other biomaterials like alginate, nanocellulose enhances cellular viability, tensile strength, and proliferation, addressing common challenges in tissue engineering.^[319]^

In cosmetic applications, nanocellulose, particularly in the forms of CNF and BNC, has emerged as an eco‐friendly ingredient for various formulations. Its ability to stabilize emulsions and efficiently deliver active ingredients has made it valuable for products ranging from creams to transdermal patches.^[320]^ The material's natural properties contribute to improved product performance while maintaining environmental sustainability.

The extraction of nanocellulose from various waste sources, including silk, sugarcane bagasse, and pineapple leaves, demonstrates the material's potential for sustainable production.[^321^, ^322^, ^323^] This approach aligns with circular economy principles, transforming waste materials into valuable resources for healthcare applications. The ability to tailor nanocellulose properties through various modification techniques further enhances its versatility and effectiveness across different biomedical applications.

As research continues to advance, the integration of waste‐derived nanocellulose in biomedical applications represents a promising intersection of sustainability and healthcare innovation. The material's natural properties, combined with its ability to be modified for specific applications, position it as a valuable resource in the development of next‐generation medical solutions. This review explores the various biomedical applications of waste‐derived nanocellulose, examining its properties, production methods, and potential impact on sustainable healthcare practices.

### 4.1 Wound Healing

Nanocellulose extracted from agricultural and forestry by‐products provides a sustainable and efficacious means for wound management. Its essential features, such as significant moisture retention, mechanical robustness, and adaptability, make it particularly advantageous for sophisticated wound dressings. Moreover, its capability to integrate bioactive agents and biopolymers promotes healing, mitigates infection risk, and fosters a more sustainable healthcare paradigm. The distinctive attributes of this nanoscale cellulose derivative present it as a potential candidate for enhancing wound care. The process of wound healing is intricate, comprising several distinct phases. Initially, hemostasis transpires at the wound site, succeeded by an inflammatory phase that endures from 24 hours to 4–6 days.^[59]^ In this phase, immune cells release proteolytic enzymes and proinflammatory cytokines, generating reactive oxygen species (ROS) to prevent infection and remove foreign bodies and debris. Concurrently, cytokines and enzymes stimulate fibroblast and myofibroblast growth, while wound exudate maintains necessary moisture for healing. The proliferation stage ensues, characterized by the formation of granulation tissue and the development of a new extracellular matrix. Finally, remodeling occurs, marked by changes in matrix composition and the replacement of collagen III with collagen I, enhancing tissue strength. Skin wound repair is intricate and influenced by various factors. Self‐healing is slow and vulnerable to infections, necessitating suitable wound dressings to facilitate and guide the process. Optimal dressings maintain high wound humidity, remove excess exudates, are non‐toxic and non‐allergenic, allow oxygen exchange, prevent microbial invasion, and are comfortable and cost‐effective. Natural and synthetic polymers are utilized for wound dressings, with natural polymers favored for their biocompatibility, biodegradability, and physicochemical properties. Biopolymer‐based dressings, designed to degrade synchronously with wound healing, ensure effective substance release. Various forms of wound dressings, such as bandages, hydrogels, films, sponges, foams, and nanofiber mats, have been developed.^[313]^

**Figure 8:**
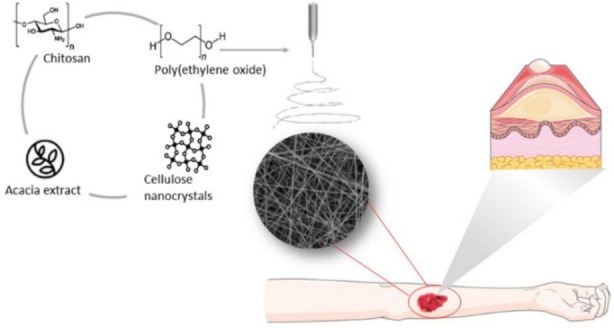
Nanocellulose preparation methods and wound dressing applications.[314] Copyright 2021, Elsevier Ltd.

Bio‐based nanofibers were successfully developed using electrospinning with chitosan, poly(ethylene oxide) (PEO), CNC, and acacia extract. Optimized parameters (2 % CS/PEO, 50 % acetic acid, 29 kV voltage, 170 mm needle‐collector distance, and 4 mL/h flow rate) produced defect‐free nanofibers with an 80 nm diameter. CNC incorporation (0.1–2 %) improved thermal stability and uniformity, while acacia extract yielded 83 nm nanofibers with enhanced antibacterial and antifungal properties. The nanofibers were shown to be biocompatible, and a sustained extract release was observed over 24 hours, making them promising for wound dressing applications (Figure 8).^[314]^

A pivotal advantage of nanocellulose‐based wound dressings lies in their moisture maintenance at the wound site, crucial for healing. The porous nanocellulose structure, resembling the skin's extracellular matrix, facilitates the supply of growth factors, macrophage movement, and fibroblast proliferation. Additionally, nanocellulose dressings effectively absorb exudates and dead tissue molecules, creating a conducive environment for wound healing.^[59]^

The versatility of nanocellulose is evident in its combination with other biopolymers, enhancing overall wound dressing performance. Bacterial cellulose, a highly pure and porous nanocellulose form produced by bacteria, reinforced with natural extracts like propolis, displayed promising wound healing properties.^[324]^ Bacterial cellulose composites immobilized with beneficial bacteria like Bacillus subtilis demonstrated resistance against pathogens.^[325]^

Researchers have explored various waste sources for extracting nanocellulose, promoting sustainability and cost‐effectiveness. For instance, CNF extracted from coconut fibers, functionalized with pomegranate extract and gold nanoparticles, exhibited significant antibacterial activities and low cytotoxicity.^[326]^

Incorporating nanocellulose into biopolymers like chitosan and collagen further enhances wound dressing properties. Wound dressing developed by impregnating bacterial cellulose fibers with collagen and chitosan, resulting in larger pore sizes, better air exchange (up to 75.5 % porosity), moisture retention (up to 99.6 %), and antibacterial properties against *S. aureus* and *E. coli*.^[327]^ Recent advances in nanocellulose‐based wound dressings have yielded promising results from various waste sources.^[328]^

Table 8 succinctly summarizes key findings from diverse studies, emphasizing the impact of nanocellulose from waste materials on wound healing applications.

**Table 8:** Nanocellulose Impact on Wound Healing from Waste Material Studies.

Waste Materials	Key Findings
Coconut fibers and functionalized with pomegranate extract and gold^[326]^	– CNF from coconut fibers, impregnated with pomegranate extract and gold nanoparticles (AuNPs), demonstrated significant antibacterial activity, reducing *S. aureus*, *E. coli*, and *P. aeruginosa* colonies by 60–99 %. The biohybrid material, characterized for stability and non‐cytotoxicity, holds substantial potential for cost‐effective antibacterial and industrial applications.
Green and red propolis^[324]^	– Bacterial cellulose‐based wound dressings, incorporating 2–4 % green and red propolis extract, exhibited variable properties: transparency (28.59–110.62T_600_ mm^−1^), thickness (0.023–0.046 mm), swelling index (48.93–405.55 %), water vapor permeability rate (7.86–38.11 g m^2^ day^−1^), elongation (99.13–262.39 %), and antioxidant capacity (21.23–86.76 μg mL^−1^). The BNC and red propolis dressing uniquely showed antimicrobial activity, suggesting potential for diverse dermal lesions.
Collagen and chitosan^[327]^	– BNC‐based wound dressings, impregnated with chitosan (Chi) and collagen (Col), demonstrated enhanced properties: FTIR confirmed intermolecular interactions, XRD showed broader peaks at 14.2°, 16.6°, and 22.4°, SEM revealed successful penetration, and BNC/Col/Chi exhibited larger zones of inhibition against S. aureus and E. coli.
Styela clava^[329]^	– Nanocellulose from Styela clava tunics, specifically freeze‐dried SCT‐CM (FSCT‐CM), demonstrated outstanding physical properties, including high tensile strength (1.64 MPa), low elongation (28.59 %), and low water vapor transmission rate (WVTR). In surgically wounded SD rats, FSCT‐CM treatment resulted in lower wound area and scores, significant histopathological improvement, stimulated collagen‐1 expression, and activated the TGF‐β1 signaling pathway, suggesting accelerated wound healing without inducing toxicity.
Wood‐derived cellulose^[330]^	– In clinical trials on burn patients, wood‐based nanofibrillar cellulose (NFC) wound dressing exhibited faster epithelialization (11–21 days) compared to Suprathel® (16–28 days). NFC, with lower content, showed biocompatibility, easy attachment, and self‐detachment, making it promising for skin graft donor site treatment.
Pinus radiata^[331]^	– Pinus radiata‐derived nanocellulose exhibited antibacterial efficacy against Pseudomonas aeruginosa PAO1 in in vitro studies. The nanocellulose films showed <4 % residual fibers, impaired biofilm growth, increased cell death, and a markedly different morphology of PAO1 compared to the commercial control wound dressing, Aquacel®.
Pseudomonas aeruginosa^[332]^	– Pseudomonas aeruginosa‐derived nanocellulose (CNF, <20 nm) exhibited dose‐dependent inhibition of bacterial growth, reduced biofilm biomass on aerogels, and unaffected virulence factor production, showcasing its potential for novel wound dressings.
Hibiscus cannabinus^[333]^	– Hibiscus cannabinus‐derived nanocellulose incorporated into Chitosan/Poly (vinyl pyrrolidone) composites (CPN) showed promising wound healing potential, with enhanced antibacterial activity (especially in CPN_3_ %), maintained moist environment, improved swelling, blood compatibility, and reduced cytotoxicity in normal mouse embryonic fibroblast cells.

### 4.2 Drug Delivery

**Figure 9:**
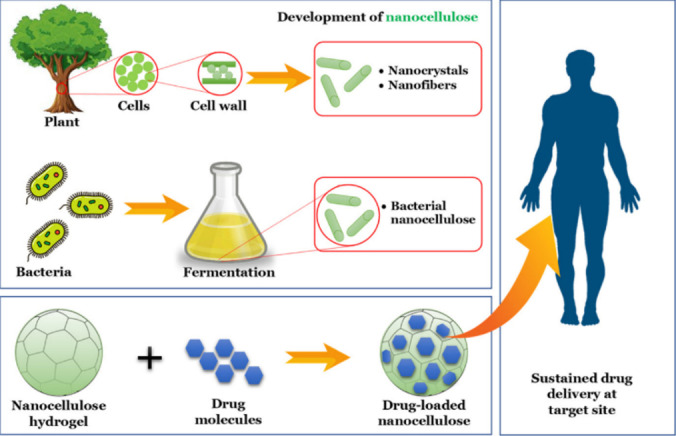
Diverse Drug Delivery.[334] Copyright 2021, Elsevier Ltd.

Nanocellulose, recognized for its extensive surface area, biocompatibility, and adjustable surface characteristics, possesses considerable promise as a vehicle for drug delivery. Biomass waste, encompassing agricultural and forestry by‐products, serves as an exemplary source of nanocellulose. The extraction of nanocellulose from such waste materials confers both environmental and sustainability benefits.^[315]^ Figure 9 provides an overview of diverse drug delivery systems, including sustained drug delivery.^[334]^

A modification method involving the grafting of chitosan oligosaccharide (CSOS) onto CNC revealed successful grafting, with drug loading efficiency of 14 % w/w and a binding efficiency of 21.5 % at pH 8. In vitro studies showed rapid drug release within 1 hour. CNC–CSOS particles, being biocompatible and biodegradable, are promising for wound dressings and local drug delivery, particularly in periodontal treatments.^[335]^

Soy protein isolate‐CNC nanoparticles exhibited sustained release of curcumin over 24 hours in a simulated gastrointestinal model. At a 6 : 1 SPI‐to‐CNC mass ratio, the nanoparticles showed a size of 197.7±0.2 nm, low polydispersity (0.14), and high encapsulation efficiency (88.3 %), with stable release across various conditions.^[336]^ In cancer applications, 5‐fluorouracil‐loaded CNC nanoparticles showed dose‐dependent toxicity in colorectal cancer cells, with 80 % drug release after 36 hours via swelling, dissolution, and diffusion mechanisms.^[50]^ Nanocellulose has also been used in various drug formulations, including tablets, aerogels, hydrogels, and membranes, offering flexible release times and diverse applications for drug delivery.^[316]^ Table 9 compiles significant discoveries from various research endeavors, highlighting the influence of utilizing nanocellulose sourced from waste materials in drug delivery applications.

**Table 9:** Nanocellulose Impact on drug delivery from waste Material Studies.

Waste Materials	Key Findings
Chitosan oligosaccharide^[335]^	– Particles exhibited 21.5 % binding efficiency, 14 % w/w drug loading, and rapid in‐vitro release within 1 hour at pH 8.
Chitosan^[337]^	‐ CNC, oxidized CNC, and chitosan oligosaccharide grafted CNC demonstrated potential as drug delivery carriers, with IMI exhibiting higher binding affinity than PrHy. Interaction types were elucidated via Isothermal Titration Calorimetry (ITC), and drug release correlated with CNC‐drug interactions.
Chitosan^[338]^	– Chitosan‐microcapsules/starch blend film enhances drug release with improved thermostability, mechanical properties, water resistance, and sustained, pH‐sensitive behavior, exhibiting pharmacodynamic efficacy.
Soy protein^[336]^	– Soy protein isolate CNC nanoparticles (197.7±0.2 nm, 0.14 PDI) at a 6 : 1 mass ratio exhibited high encapsulation efficiency (88.3 %) and sustained curcumin release, showcasing potential for hydrophobic compound delivery.
Egg white protein^[339]^	– Egg white protein nanoparticles at pH 3.0 achieved the highest curcumin loading of 11.53 mg/g, exhibiting effective preservation of curcumin's antioxidant activity.
Whey protein^[340]^	– Whey protein microgels loaded with curcumin showed a loading amount of 17.51±0.46 μg curcumin/mg protein, increased diameter, altered surface charge, reduced sedimentation, amorphous curcumin encapsulation, and slower release during simulated gastrointestinal conditions.
Zein^[341]^	– Pectin and CMC‐based cross‐linked nanoparticles (160–210 nm) exhibited superior stability, spherical morphology, 80 % encapsulation efficiency, slower release, and improved antioxidant activity, showcasing potential for oral delivery applications.
Cotton wool^[342]^	– Hhigh gene transfection efficiency, low cytotoxicity, and potent in vivo antitumor activity, showcasing their promising role in biomedical applications.
Mucilage^[343]^	– Mucilage/HPMC transdermal patches exhibited prolonged drug release, skin irritation‐free characteristics, and no significant interactions, suggesting their potential as effective drug delivery devices.
Pectin, Gelatin^[344]^	– Pectin‐gelatin hydrogel blend patches, utilizing a two‐step gelation procedure, demonstrated tunable rheological and pharmaceutical characteristics for transdermal drug delivery, with detailed assessments of structural properties and in vitro release profiles.

### 4.3 Biosensing

**Figure 10:**
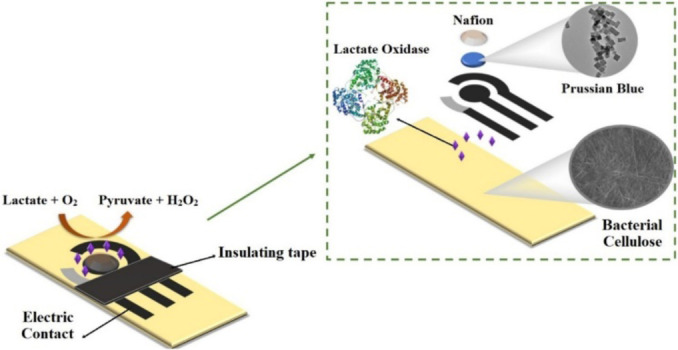
Wearable Lactate Detection Biosensor with BNC Substrate.[345] Copyright 2021, Elsevier Ltd.

Biosensors, integral for health monitoring and diagnostics, are advancing towards portable wearable devices that offer convenience, portability, and minimal sample preparation. Waste‐derived nanocellulose has gained attention as a sustainable material for biosensing applications, providing efficient and cost‐effective solutions. Nanocellulose‐based platforms, such as hydrogels and aerogels, can be designed to produce various responses, including colorimetric, photoluminescent, mechanical, and electrical, making them adaptable for different biosensing mechanisms. Due to its renewable, low‐cost nature, nanocellulose presents an eco‐friendly alternative to traditional sensing platforms, particularly in applications like filaments, paper‐based platforms, and gel‐like sensing devices, showing significant potential for future innovations.^[317]^ For health monitoring, a smart wearable sensor using BNC as a substrate was developed to detect lactate in artificial sweat (Figure 10). The sensor, which immobilized lactate oxidase directly onto a Prussian blue nanocubes‐modified carbon electrode, demonstrated effective lactate detection in concentrations ranging from 1.0 to 24.0 mmol L^−1^.^[345]^

Wearable devices such as rings, patches, wristbands, and tattoos have been created using nanocellulose nanocomposites, offering advantages in portability, ease of use, and continuous health monitoring. A sensitive enzymatic biosensor for cholesterol, using PANi/CNC/IL nanocomposites, has also been developed, demonstrating excellent stability and reproducibility for cholesterol monitoring in food and clinical settings.^[346]^ Waste‐derived nanocellulose from Semantan bamboo was utilized to create a highly sensitive cholesterol biosensor. The biosensor, incorporating cholesterol oxidase on a PANi/CNC/IL/SPE electrode, exhibited outstanding performance. Ionic liquid enhanced electron transfer, validated by imaging techniques. Research Surface Methodology optimization yielded a sensitivity of 35.19 μA mM/cm^−2^. The biosensor displayed a broad linear range (1 μM to 12 mM, R^2^=0.99083) and a low 0.48 μM Limit of Detection. Exceptional reproducibility (RSDs ≤3.76 %) and minimal interference with coexisting electroactive compounds highlight the eco‐friendly innovation, showcasing the potential for sustainable cholesterol monitoring.^[347]^

A proficient amperometric biosensor utilizing waste‐derived nanocellulose from chitosan has been developed for phenol detection. Immobilizing tyrosinase enzyme in an CNC/chitosan composite film showcased advantages such as minimal sample preparation, selectivity, sensitivity, and rapid response time. The biosensor demonstrated a linear response within the phenol concentration range of 0.39–7.74 μM, with a detection limit of 0.379 μM. Excellent reproducibility (4.27 % R.S.D at 50 μM phenol) showcased its reliability for quantifying phenolic compounds in industrial settings, presenting a promising advancement in biosensor technology.^[348]^

Waste‐derived nanocellulose from cotton was employed in a biosensor for elastase detection, a biomarker linked to inflammatory diseases like chronic wounds. CNC/chitosan composite films exhibited eco‐friendly and biocompatible properties. Computational modeling elucidated the nanocrystalline surface's features, showcasing efficient ligand‐protease interaction. The peptide‐conjugated nanocrystal model showcased elastase docking accessibility. Experimental results highlighted a 30‐fold increase in elastase binding affinity compared to a freely soluble peptide. Both colorimetric and fluorescent responses demonstrated the biosensor's sensitivity, presenting potential for point‐of‐care diagnostics, particularly in wound management applications.^[349]^ Table 10 summarizes studies on the impact of nanocellulose derived from waste materials on biosensor performance.

**Table 10:** Nanocellulose Impact on biosensor from waste Material Studies.

Waste materials	Key Findings
Bamboo^[347]^	– The biosensor showcased a sensitivity of 35.19 μA mM/cm^−2^, a broad linear range (1 μM to 12 mM, R^2^=0.99083), and a 0.48 μM Limit of Detection, highlighting outstanding reproducibility (RSDs ≤3.76 %).
Cotton^[348]^	– A biosensor for elastase detection showed a 30‐fold increase in binding affinity, with both colorimetric and fluorescent responses demonstrating sensitivity for potential point‐of‐care diagnostics in wound management.
Chitosan^[350]^	– The biosensor exhibited a linear response in the range of 0.39–7.74 μM, with a low Limit of detection of 0.379 μM, and demonstrated excellent reproducibility (4.27 % R.S.D at 50 μM phenol).

### 4.4 3D‐Bioprinting

Bioprinting methodologies, encompassing inkjet, laser‐assisted, and extrusion‐based techniques, have markedly progressed the field of tissue engineering by introducing complexities necessitating bioinks with tailored rheological attributes.^[318]^ The mechanical integrity of bioinks is pivotal for fabricating tissues that mimic the properties of native tissues. Nanocellulose, sustainably sourced from byproducts, has emerged as an exemplary bioink owing to its superior rheological properties, biocompatibility, and sustainability.^[351]^

Nanocellulose exhibits considerable potential in 3D bioprinting applications for diverse tissues, including blood vessels, bone, cartilage, skeletal muscle, neural, dermal, and vascular scaffolds. When integrated with alginate, a frequently utilized biomaterial, it mitigates issues such as inadequate cross‐linking and moderate cellular adhesion, resulting in enhanced cell viability, tensile strength, and cellular proliferation. Nanocellulose‐alginate bioinks have evidenced remarkable tissue viability, precise printing fidelity, and improved cellular proliferation.^[319]^

Incorporating CNC obtained from wood pulp into 3D bioprinting through a digital light processing (DLP) approach, blending CNC with poly(ethylene glycol) diacrylate (PEGDA) and 1,3‐diglycerolate diacrylate (DiGlyDA) improved CNC compatibility with the PEGDA matrix. Analysis using the Halpin–Tsai model and polarized light microscopy confirmed effective CNC dispersibility. Mechanical testing demonstrated enhanced properties of DLP 3D printed composites with CNC and also highlighted the influence of curing layer thickness on mechanical and water swelling properties. The versatile approach showcased the successful creation of complex 3D CNC composites with improved properties, offering potential advancements in cellulose‐based applications for 3D bioprinting. CNC dimensions were characterized through TEM analysis, indicating their suitability for various applications.^[352]^

The acetylated nanocellulose bioinks, derived from birch wood fibers, demonstrated shear‐thinning behavior for direct ink writing (DIW), allowing efficient extrusion during the printing process. In comparison to unmodified and TEMPO‐oxidized nanocelluloses, the acetylated nanofibrils required a significantly lower concentration to achieve bioinks with similar performance, resulting in more porous structures. The bioinks exhibited enhanced stability during drying and rewetting processes, ensuring dimensional stability for packaging and sterilization. Moreover, the acetylated nanocellulose bioinks produced monolithic scaffolds with a high surface charge and axial aspect, facilitating cellular attachment, proliferation, and viability of cardiac myoblast cells for a duration of 21 days. These findings highlight the potential of acetylated nanocellulose bioinks for scalable fabrication of scaffolds suitable for tissue engineering applications. The data indicate superior performance and biocompatibility, showcasing the versatility of acetylated nanocellulose in advancing 3D bioprinting technologies.^[353]^

By incorporating CNC derived from the abaca plant, known for its superior strength and stiffness, to enhance mechanical and surface properties, the study successfully demonstrated 3D printing of a CNC nanocomposite hydrogel through stereolithography (SL). This process formed intricate structures suitable for tissue engineering. Thermal analysis revealed that the nanocomposite hydrogels exhibited excellent stability up to 400 °C, surpassing traditional methods.^[354]^

Bagasse‐derived fibers, sourced from Qena Pulp and Paper Industry in Egypt, underwent TCNF. The carboxylate content of T‐CNF was determined as 1.2±0.4 mmol g^−1^ through conduction volumetric analysis. Chemical products, including EMPO, sodium hypochlorite, sodium hydroxide, sodium chlorite, acetic acid, and sodium bromide, were obtained from Sigma‐Aldrich. This study delves into the application of waste‐derived nanocellulose from bagasse in 3D bioprinting, focusing on its potential to fabricate intricate structures with superior material properties, particularly beneficial for tissue engineering. The results showcase a substantial 20.1 % hydroxyapatite mineralization, indicating enhanced cell attachment. Additionally, the introduction of CNF significantly improved the viscosity recovery of alginate hydrogel, leading to a more resilient composite scaffold with enhanced structural integrity during both the printing and biomimetic mineralization processes.^[355]^ Table 11 provides key findings on the influence of nanocellulose sourced from waste materials on studies related to 3D‐bioprinting.

**Table 11:** Nanocellulose Impact on 3D‐Bioprinting from waste Material Studies.

Waste materials	Key Findings
Silkworm silk^[356]^	– Enhanced shear thinning in various polymer solutions for 3D bioprinting. Cell viability remained high (92.5–92.7 %) with SFNFs, showcasing biocompatibility and printability improvements.
Wood pulp^[352]^	– Blending with DiGlyDA improved compatibility. Halpin–Tsai analysis and microscopy confirmed effective CNC dispersibility. Mechanical tests showed enhanced properties.
Birch wood fiber^[353]^	– Enhanced stability, cellular attachment, proliferation, and viability were observed for cardiac myoblast cells, highlighting biocompatibility and versatility.
Hardwood kraft pulp^[357]^	– Optimized through material characterization and printing experiments, exhibiting enhanced mechanical properties, biofunctionalization capabilities, and potential applications in tissue‐compatible devices and wearable sensors.
Abaca pulp^[354]^	– Increased durability and strength of printed constructs through CNC loading. Enhanced thermal stability achieved with CNC up to approximately 400 °C.
Bagasse^[355]^	– Enhanced material properties, achieving 20.1 % hydroxyapatite mineralization, and improved alginate hydrogel viscosity recovery for robust scaffolds.

### 4.5 Cosmetic

Nanocellulose, particularly CNF and BNC, has gained attention as an eco‐friendly and effective ingredient in cosmetic formulations. Its key role lies in stabilizing emulsions in products such as creams, lotions, and fragrance delivery systems through Pickering stabilization.^[320]^ Nanocellulose's ability to load and release cosmetic active ingredients efficiently is another significant advantage. For instance, microbial nanocellulose used in skin tanning products successfully absorbed dihydroxyacetone (DHA), releasing it gradually over time and preventing issues such as leaching and unpleasant odors often associated with traditional DHA creams. Additionally, flexible nanocels show potential as substrates for transdermal patches, enabling the controlled delivery of cosmetic ingredients like skin whiteners and anti‐aging treatments.

The BioMask, integrating BNC and 2 % propolis extract, emerges as a promising solution for dermatological and cosmetic applications. Produced in Hestrin‐Schramm (HS) medium, the BNC film showcases hypoallergenic, non‐toxic, highly water‐retentive, and biocompatible properties, validated by XRD and TGA spectroscopy. Propolis infusion, rich in polyphenols and flavonoids, enhances its medicinal potential. This BioMask prototype, tailored for acne‐related skin inflammations, highlights B‐ nanocelluloses hydration benefits, resulting in pain reduction, improved skin texture, and accelerated healing with propolis. Offering immediate relief and disposal, it contributes to enhanced self‐esteem for acne sufferers, showcasing BNC and propolis’ versatility in sustainable skincare solutions.^[358]^

Nanocellulose derived from waste Pili (Canarium ovatum) pulp emerges as a sustainable and environmentally friendly substitute for mineral‐based cosmetics. Employing conventional cellulose extraction techniques, including boiling and alkalization, resulted in successful isolation of cellulose from lignocellulosic and hemicellulosic materials. Sulfuric acid hydrolysis at 64 wt % and varying reaction times (30, 45, and 60 minutes) produced micro‐sized cellulose with an average particle size of 3.4×105 nm, showcasing cellulose purity with an 85 % match to α‐cellulose in infrared spectrum analysis. Surface morphology analysis revealed nanostructures, while UV‐Vis Spectrophotometer results indicated absorbances at 278 nm, increasing with prolonged hydrolysis.^[359]^ Table 12 presents insights into the impact of utilizing nanocellulose derived from waste materials in studies focusing on cosmetic applications.

**Table 12:** Nanocellulose Impact on Cosmetic application from Waste Material Studies.

Waste materials	Key Findings
Bamboo^[360]^	– Unbleached bamboo waste nanocellulose enhances stability in polyethylene emulsions (0.3 % w/w) under varied pH (3.0–9.0) and temperatures (4–50 °C).
Propolis^[358]^	– The BioMask, blending BNC with 2 % propolis extract, is a potent dermatological solution for acne. BNC film's hypoallergenicity, non‐toxicity, and water retention are confirmed. Propolis, rich in polyphenols, enhances medicinal properties.
Waste Pili (Canarium ovatum) pulp^[359]^	– Synthesized through 64 % sulfuric acid hydrolysis, exhibits promising optical properties, suggesting sustainability as a cosmetic ingredient.

## 5 Conclusions

The production of nanocellulose from waste biomass sources involves a systematic sequence of pretreatment, treatment, and post‐treatment processes. Pretreatment methods like alkaline, acid, and oxidative treatments play a crucial role in removing impurities and enhancing cellulose fiber accessibility. Subsequently, the treatment phase employs mechanical disintegration techniques (Grinding, Cryocrushing, Homogenization, Ultrasonication, Steam explosion, TSE, Non‐Covalent treatment, Radiation, Electrospinning, Aqueous counter‐collision) coupled with chemical processes (Acid hydrolysis, TEMPO‐mediated oxidation, Bleaching, Mercerization, Esterification, Acetylation, Etherification, Hydrolysis with metal salt catalysts, Periodate oxidation, Reduction, Silanization, Grafting, Electrochemical Processes/ Plasma) to isolate nanocellulose from the treated fibers. Biological methods like enzymatic hydrolysis and genetic modification present sustainable alternatives for nanocellulose production. Post‐treatment processes, such as surface coating, crosslinking, and drying, further tailor nanocellulose properties for specific applications, enhancing characteristics like mechanical strength, solubility, and stability.

Various techniques enable the manufacture of nanocellulose from waste materials, showcasing its versatility. Optimizing pretreatment, treatment, and post‐treatment processes allows customization of nanocellulose properties, particularly for biomedical applications. Integrating waste biomass aligns with circular economy principles, promoting resource efficiency and driving innovation for sustainable nanocellulose production.

Biomedical applications of nanocellulose from waste sources show promising results, indicating its potential in healthcare innovation. Nanocellulose wound dressings exhibit high moisture retention and antibacterial properties, fostering faster healing and infection prevention. For instance, CNF infused with pomegranate extract and gold nanoparticles reduce bacterial colonies by 60–99 %. Nanocellulose drug carriers, like chitosan‐grafted CNC particles, ensure rapid drug release within 1 hour at pH 8, with 21.5 % binding efficiency and 14 % w/w drug loading, offering sustainable solutions for healthcare challenges.

Electrochemical biosensors with nanocellulose thin films show impressive sensitivity and selectivity, detecting adenine and guanine bases with low limits of detection (1.4×10^−6^ mol L^−1^ and 1.7×10^−7^ mol L^−1^, respectively). Wearable biosensors employing nanocellulose nanocomposites offer advantages for continuous health monitoring. Enzymatic biosensors utilizing waste‐derived nanocellulose exhibit exceptional sensitivity (35.19 μA mM/cm^−2^) in cholesterol detection. Amperometric biosensors with nanocellulose from chitosan reliably detect phenolic compounds.

Waste‐derived nanocellulose is a promising bioink for 3D bioprinting, enhancing tissue engineering with its rheological properties, biocompatibility, and sustainability. Studies consistently demonstrate nanocellulose ‐based bioinks’ efficacy in fabricating intricate tissue structures with enhanced mechanical properties and cellular viability. Combining nanocellulose with alginate synthesizes vascularized bone tissue, showcasing its potential in bone tissue engineering, while successfully constructing various tissues underscores nanocelluloses versatility. These findings highlight NC's transformative potential in advancing 3D bioprinting for tissue engineering, offering sustainable solutions for biomedical applications.

Nanocellulose proves its value in cosmetics, as seen in its ability to stabilize emulsions, extending product shelf‐life and efficacy. Studies confirm its efficacy in preventing coalescence and creaming, ensuring product stability over time. Its high surface area allows for efficient loading and sustained release of active ingredients, enhancing skincare benefits while minimizing adverse effects. For example, waste‐derived nanocellulose from bamboo and Pili pulp demonstrates stability and suitability for cosmetics.

## Biographical Information

*Dr. Mehrdad Ghamari is a distinguished researcher specializing in waste‐derived nanocellulose and green energy. With a robust background in structural engineering, he excels in numerical modeling for building structures and pioneering cooling methods utilizing waste‐derived materials, significantly advancing energy efficiency in construction. His innovative contributions, underpinned by a commitment to sustainability and global recognition, highlight a remarkable track record of impactful research and expertise in sustainable technologies*.

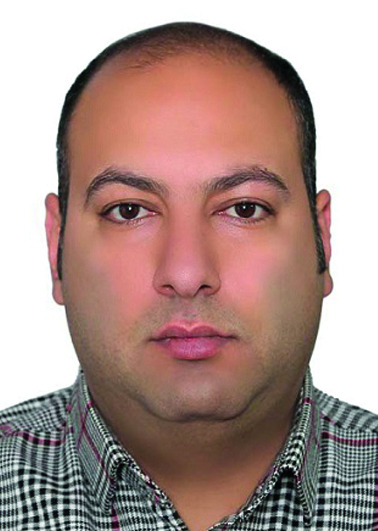

## Biographical Information

*Dr. Suvish, a physician trained in Medicine at Manipal University with a residency in Neurology from the Institute of Human Behaviour & Allied Sciences (IHBAS), also holds a Master's in Public Health from Glasgow Caledonian University, UK. Currently pursuing a PhD in Medical Engineering at Teesside University, his research focuses on the development of innovative biomedical sensors for assistive technological applications. His work within the neurodegenerative disease spectrum emphasizes the design, development, and optimization of novel biosensors capable of accurately detecting early bio‐signal changes, that are indicative of subtle degenerative changes in daily living*.

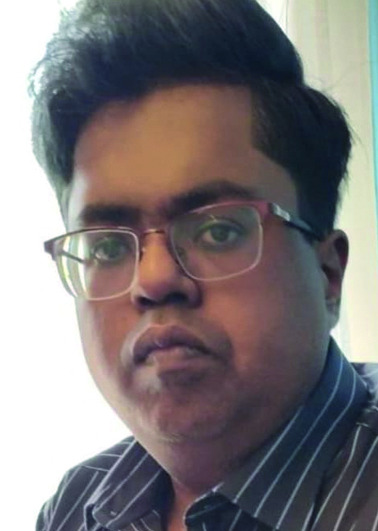

## Biographical Information

*Prof. Chan H. See, a Professor at Edinburgh Napier University, holds a first‐class B.Eng. Honours and a Ph.D. from the University of Bradford. Formerly head of Electrical Engineering & Mathematics, his teaching focuses on embedded systems, communication electronics, and IoT. His research includes IoT, wireless sensor networks, microwave sensors, and antennas, with over 150 peer‐reviewed articles. Recognized in Stanford University's World Top 2 % Scientists list, he has received the IEEE Malaysia AP/MTT/EMC Joint Chapter Best Paper Award and two Young Scientist Awards. A Chartered Engineer and Fellow of IET, he also serves as an Associate Editor for IEEE Access*.

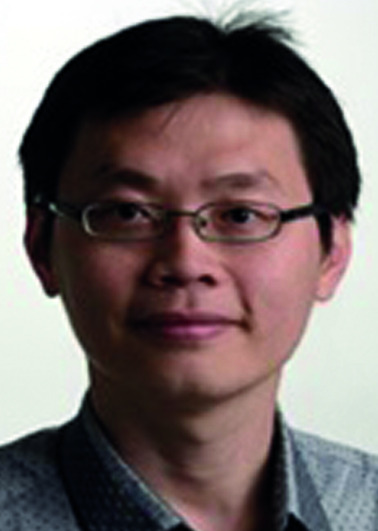

## Biographical Information

*Prof. Hongnian Yu, a Professor at Edinburgh Napier University, has supervised 26 PhD and 20 Master's theses and trained 12 post‐doctoral fellows. With over 200 research papers and research grants totaling around ten million pounds, his expertise covers robotics, intelligent control, applied AI, data analysis, and technology applications in healthcare and manufacturing. He has led international projects funded by EPSRC and the EU, collaborating with partners from over 30 countries. Prof. Yu holds fellowships in IET, RSA, and senior membership in IEEE, earning numerous awards and medals*.

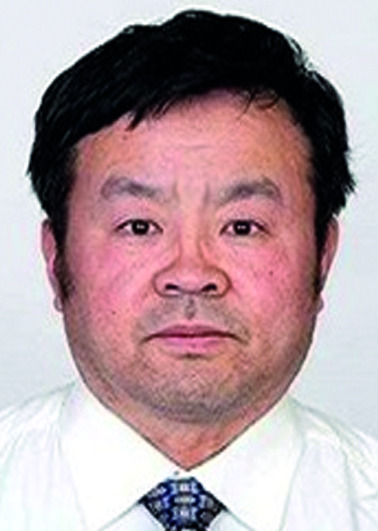

## Biographical Information

*Dr. Anitha Thiyagarajan holds a Ph.D. in Biochemistry from Bharathiyar University, specializing in medicinal plants as therapeutics. She began her research career studying HIV infection and antiretroviral therapy in collaboration with Dr. M.G.R. Medical College. As a senior research fellow at Tamil Nadu Agricultural University, she worked on genetic transformation of Jatropha curcas. Currently, she is an Assistant Professor of Biochemistry at Horticultural College, Periyakulam, managing five externally funded projects. A published researcher in international journals, she is a recipient of prestigious awards, including the Dr. E.K. Janaki Ammal Best Women Scientist Award*.

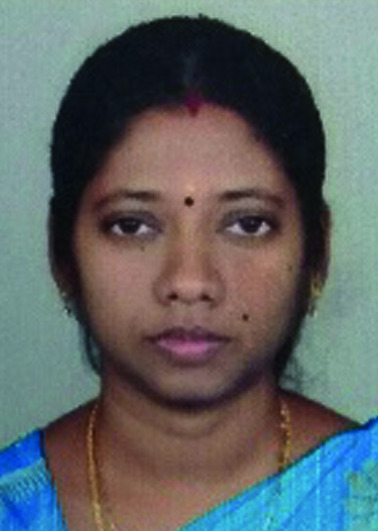

## Biographical Information

*Prof. Balamurugan, Professor and Head at Bannari Amman Institute of Technology, holds a B.Tech from VIT (2003) and a Ph.D. in Biomedical and Computer Science Engineering from the University of Luebeck, Germany. A visionary leader, he specializes in translating research into products, mentoring teams, and fostering academia‐industry collaboration. Previously a Research Consultant at Robert Bosch, Pricol, and Matrimony.com, his expertise spans AI, Machine Learning, IoT, and biosensors. With four patents and multiple international publications, he combines technical excellence with a passion for music, driving innovation and entrepreneurship*.

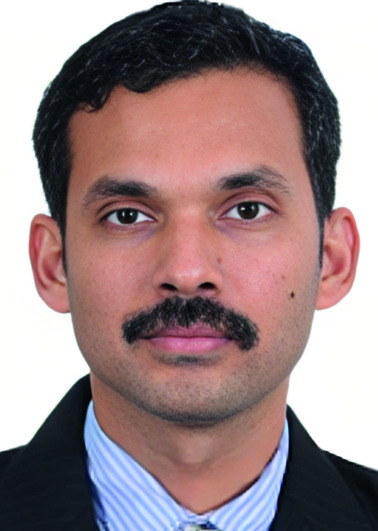

## Biographical Information

*Dr. Sasireka Velusamy is a research associate at Teesside University, currently working on an innovative project focused on hydrogen production from biomass agricultural waste. She completed her PhD at the University of Exeter and has a strong background in wastewater treatment. Her research has involved the use of graphene membranes to remove contaminants, especially heavy metals, from industrial wastewater, making significant contributions to sustainable solutions aimed at reducing the environmental impact from industrial wastewater. She received her MPhil degree from Bharathidasan University, India, and her Master's degree from Bharathiar University, India*.

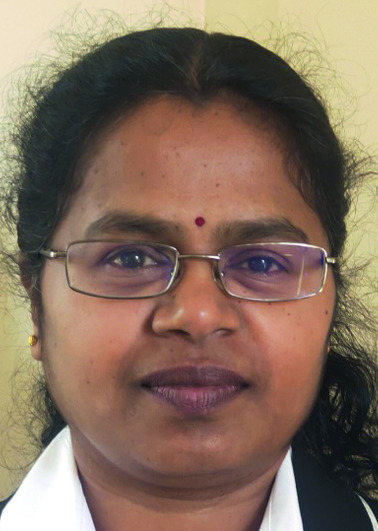

## Biographical Information

*Prof. David Hughes is the Associate Dean for Research & Innovation at Teesside University, leading the NetZero research theme and the Circular Economy and Recycling Innovation Centre (CERIC). An expert in the circular economy, he specializes in industrial digitalization, AI, and machine learning solutions for heavy industry. His work focuses on low‐carbon materials and sustainable trade models enabled by digital technologies. He collaborates with industry to valorize by‐products, advancing SDG13 (climate action) and SDG12 (responsible production). As Chair of the IOM3 Polymer Group and a member of government steering groups on plastics and cement, he drives innovation in circularity and sustainability*.

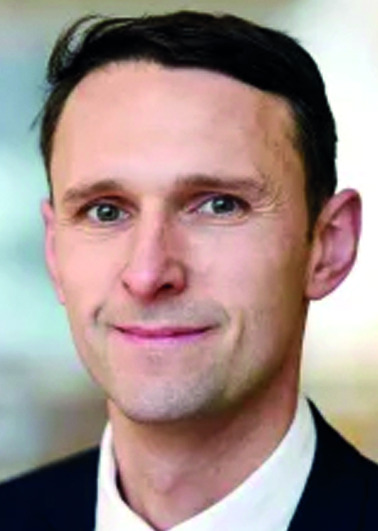

## Biographical Information

*Prof. Senthilarasu Sundaram, a Professor at Teesside University's School of Computing, Engineering, and Digital Technologies, specializes in sustainable energy, particularly solar energy. His research journey began during his MPhil in Applied Physics and led to a PhD focused on organic solar cell materials. Prof. Sundaram has expertise in resource assessment, theoretical modeling, and simulation of various solar systems, including thin‐film photovoltaics and third‐generation solar cells. His work involves sustainable energy generation using environmentally friendly materials such as dye‐sensitized, tin‐based perovskite, and thin‐film solar cells, focusing on energy generation, storage, and low‐carbon applications for diverse sectors*.